# How CD40L reverse signaling regulates axon and dendrite growth

**DOI:** 10.1007/s00018-020-03563-2

**Published:** 2020-06-06

**Authors:** Paulina Carriba, Alun M. Davies

**Affiliations:** School of Biosciences, Cardiff University, Museum Avenue, Cardiff, CF10 3AX Wales

**Keywords:** CD40, Reverse signaling, Axon and dendrite development, Protein kinase C, c-jun N-terminal kinase, Extracellular signal-regulated kinases, Tyrosine-protein kinase syk

## Abstract

CD40-activated CD40L reverse signaling is a major physiological regulator of axon and dendrite growth from developing hippocampal pyramidal neurons. Here we have studied how CD40L-mediated reverse signaling promotes the growth of these processes. Cultures of hippocampal pyramidal neurons were established from *Cd40*^−/−^ mouse embryos to eliminate endogenous CD40/CD40L signaling, and CD40L reverse signaling was stimulated by a CD40-Fc chimera. CD40L reverse signaling increased phosphorylation and hence activation of proteins in the PKC, ERK, and JNK signaling pathways. Pharmacological activators and inhibitors of these pathways revealed that whereas activation of JNK inhibited growth, activation of PKC and ERK1/ERK2 enhanced growth. Experiments using combinations of pharmacological reagents revealed that these signaling pathways regulate growth by functioning as an interconnected and interdependent network rather than acting in a simple linear sequence. Immunoprecipitation studies suggested that stimulation of CD40L reverse signaling generated a receptor complex comprising CD40L, PKCβ, and the Syk tyrosine kinase. Our studies have begun to elucidate the molecular network and interactions that promote axon and dendrite growth from developing hippocampal neurons following activation of CD40L reverse signaling.

**Electronic supplementary material:**

The online version of this article (10.1007/s00018-020-03563-2) contains supplementary material, which is available to authorized users.

## Introduction

The growth and elaboration of neural processes during development and maturity have a major bearing on the establishment and modification of the functional properties of neural circuits. In addition to intrinsic developmental programs in neurons and the pattern of electrical activity, a wide variety of extrinsic signals orchestrate the growth, elaboration and remodeling of axons and dendrites, including delta-notch, Eph-Ephrins, cell adhesion molecules, neurotrophins, semaphorins, and slits [1, 2]. One of the latest groups of proteins recognized to influence the growth of neural processes during development is the tumor necrosis factor superfamily (TNFSF). The 19 members of this superfamily bind to one or more members of the TNF receptor superfamily (TNFRSF) and are active as membrane-integrated ligands and as soluble ligands following cleavage from the cell membrane [3]. In addition, several TNFRSF members can also act as ligands for the membrane-integrated TNFSF to which they bind, functioning as reverse signaling receptors [4]. While the TNF and TNFR superfamilies are best understood for their many roles in the immune system [5], several members of these superfamilies act on different kinds of neurons during circumscribed phases of development, either enhancing or inhibiting the growth and elaboration of axons and/or dendrites by either forward or reverse signaling mechanisms [6–20].

Bidirectional signaling between membrane-integrated CD40 ligand (CD40L, TNFSR5) and CD40 (TNFRSF5) is a particularly important physiological regulator of axon and dendrite growth in many populations of neurons in the developing nervous system. In vivo analysis of wild type and *Cd40*^−/−^ mice together with a variety of in vitro experiments have demonstrated that CD40L-activated CD40-mediated forward signaling promotes early sensory axon growth [20], whereas CD40-activated CD40L-mediated reverse signaling promotes axon growth from subsets of sympathetic neurons [15], promotes dendrite and axon growth from hippocampal pyramidal neurons [18] and inhibits dendrite growth from striatal medium spiny neurons [18].

Given the extensive involvement of CD40-activated CD40L-mediated reverse signaling in regulating axon and dendritic growth in the developing peripheral and central nervous systems, the objective of the present study was to elucidate the key signaling networks downstream of CD40L that mediate this response in the well-characterized hippocampal pyramidal neuron model. We focused on three signaling pathways that have been shown to play important roles in regulating the growth of neural processes from a variety of neurons, namely those utilizing protein kinase C (PKC), extracellular regulated kinases 1 and 2 (ERK1/ERK2) and c-Jun N-terminal kinase (JNK). PKC is particularly relevant because activation of PKC has been implicated in mediating the effects of CD40L reverse signaling on dendrite growth from hippocampal pyramidal neurons and striatal medium spiny neurons [18]. PKC activation has also been implicated in the facilitation of axon growth by TNF reverse signaling [12, 16]. ERK1/ERK2 activation is necessary for enhanced sympathetic axon growth in response to TNF reverse signaling [12] and for enhanced sensory axon growth in response to CD40 forward signaling [20]. ERK1/ERK2 activation has also been implicated in enhanced neurite growth to various factors [9, 21–23] and suppression of ERK1/ERK2 activation contributes to the suppression of axon growth by TWE-PRIL reverse signaling [19]. The JNK family has numerous functions in the nervous system including regulating dendrite growth and architecture [24].

Here we studied the effects of specific pharmacological inhibitors and activators of PKC, ERK1/ERK2, and JNK signaling on axon and dendrite growth from cultured hippocampal pyramidal neurons. Our results have revealed that an interdependent network of signaling pathways mediate and regulate the actions of CD40L reverse signaling on the growth of axons and dendrites from hippocampal pyramidal neurons. We have also begun to identify the molecular components of the CD40L receptor complex initiated by activation of reverse signaling.

## Material and methods

### Mice

Mice were housed in a 12 h light–dark cycle with access to food and water ad libitum. Breeding was approved by the Cardiff University Ethical Review Board and was performed within the guidelines of the Home Office Animals (Scientific Procedures) Act, 1986. *Cd40* null mutant mice in a C57BL6/J background were purchased from The Jackson Laboratory (Maine, USA). These mice were backcrossed into a CD1 background. *Cd40*^+/−^ mice were crossed to generate *Cd40*^−/−^ mice from which cultures were established.

### Neuron culture

Primary hippocampal neuron cultures were prepared as described previously [25] with modifications. Briefly, hippocampi were dissected from embryonic day 18 (E18) mouse embryos and were triturated to produce a single cell suspension following trypsin digestion (Worthington, Lakewood, USA) and DNase I treatment (Roche Applied Science, East Sussex, UK). The neurons were plated in plastic dishes coated with poly-l -lysine (Sigma-Aldrich, Dorset, UK) at a density of 15,000 cells/cm^2^ for morphological analysis and of 21,000 cells/cm^2^ for western blot experiments. The neurons were cultured in complete medium that contains Neurobasal A medium (Invitrogen, Paisley, UK) supplemented with 2% NeuroCult SM1 (StemCell, Cambridge, UK), 0.5 mM GlutaMAX I (Invitrogen, Paisley, UK), 100 units/ml penicillin, and 100 μg/ml streptomycin (Gibco BRL, Crewe, UK). The cultures were incubated at 37 °C in a humidified atmosphere containing 5% CO_2_. The culture medium was partially replaced with fresh complete medium every 4–5 days.

The cultures were treated as indicated in the text with the following reagents: Fc protein (1 μg/ml [18.87 nM], ALX-203–004-C050), CD40-Fc (1 μg/ml [18.52 nM], ALX-522–016-C050), U0126 (1 μM, BML-EI282-0001), and SP600125 (1 μM, BML-EI305-0010) from Enzo Life Sciences; Go6983 (500 nM, cat. no. 2285), Fisetin (1 μM, cat. no. 5016), U0124 (1 μM, cat. no. 1868) and Anisomycin (50 nM, cat. no. 1290) from Tocris Biosciences; phorbol-12-myristate-13-acetate (PMA) (500 nM, MERCK, cat. no. 524400). Except for Fc protein and CD40-Fc that were reconstituted with sterile H_2_O, the rest was reconstituted in DMSO and subsequently diluted in culture medium to the concentrations indicated. No differences were observed between control cultures that received equivalent level of DMSO, untreated cultures, and cultures treated with Fc.

### Analysis of dendrite and axon morphology

The neurite arbors of a subset of the neurons were visualized by transfecting the neurons with a GFP expression plasmid after 7 days in vitro using lipofectamine 2000 (Invitrogen, Paisley, UK) according to the manufacturer’s instructions with modifications. Briefly, the cultures were treated for 3 h with a mixture of the expression vector and lipofectamine, after which they were cultured for a further 48 h. The neurons were then fixed for 30 min with 4% paraformaldehyde. Fluorescent neurons were visualized using a Zeiss LSM710 confocal microscope. Dendrites and axons were clearly distinguishable by their morphology, which permitted their lengths to be separately quantified [13, 25, 26]. Total dendrite length and axon length were assessed using Fiji (ImageJ) software with the semiautomated plugin Simple Neurite Tracer [27]. The mean and standard errors of the measurements from multiple neurons in at least three independent experiments were plotted.

### Prediction of protein–protein interactions with STRING

An in silico approach was used to determine possible protein–protein interactions (PPI) between CD40L and PKCβ using the STRING database (Search Tool for the Retrieval of Interacting Genes/Proteins, https://string-db.org/). Mouse CD40L and PKCβ were analyzed for all possible interactions including textmining, experiments, databases, co-expression, neighborhood, gene fusion, and co-occurrence with reliability scores more than 0.4 (where 0.4 corresponds to medium confidence). Supplemental Table 1 shows all possible PPI above at this confidence level for CD40L, PKCβ, and all that they share in common.

### Downregulation of PKCβ

The level of PKCβ protein was specifically reduced using siRNA, as previously reported [18]. Briefly, neurons were transfected using lipofectamine with Silencer Select siRNA oligonucleotides at final concentration of 10 nM. The siRNAs used were silencer select negative control n.1 and Prkcβ mouse (catalogue numbers 4390843 and s71692, respectively, Thermofisher, UK).

### CD40L pull down

For the analysis of protein interactions of CD40L after activating reverse signaling, 8 day cultures of hippocampal neurons from *Cd40*^−/−^ mice were treated for 30 min with either CD40-Fc or control Fc (1 μg/ml). The neuron cells were then washed with ice-cold PBS, harvested, and lysed in ice-cold triton lysis buffer (NaCl 150 mM, EDTA 10 mM, Tris–HCl 10 mM pH7.4, 1% Triton X-100 and protease and phosphatase inhibitor cocktail mix (Protease/Phosphatase inhibitor cocktail, 5872, Cell Signaling)). After lysate clearance by centrifugation at 4 °C for 20 min at 18,000 × *g* and quantify for equal concentration, Fc fragments were pulled down from the supernatant by incubation overnight on an orbital shaker at 4 °C with protein G-Sepharose beads (Protein G Sepharose Fast Flow, P3296, Sigma, UK) previously blocked with 5% BSA. The beads were then washed 5 × with ice-cold triton lysis buffer. Complexes were collected with 0.1 M pH 2.5 elution citrate buffer. The pH was adjusted by adding 1/6 neutralizing Tris HCl 1 M pH 8.5 buffer and after adding Laemmli buffer samples were boiled for analysis by immunoblotting.

### Immunoblotting

Dissected hippocampi from mice at 18 days embryonic (E18), postnatal at day 0 (P0), day 3 (P3), day 6 (P6), day 9 (P9) and adult (> 3 months) were placed in triton lysis buffer supplemented with protease and phosphatase inhibitor cocktail mix (protease/phosphatase inhibitor cocktail, 5872, Cell Signaling). The tissue was disaggregated using a pellet pestle until complete homogenized. For the cultured neurons from *Cd40*^−/−^ mice, after the indicated treatments neurons were scraped out of the plates in ice-cold PBS, collected by centrifugation at 4 °C for 5 min at 200 × *g* and resuspended in ice-cold triton lysis buffer. Before protein separation by immunoblotting, samples in triton lysis buffer were cleared of debris by centrifugation at 4 °C for 20 min at 18,000 × *g*. For the preparation of cytosolic and nuclear extracts, neurons were resuspended in buffer A (10 mM Hepes pH 7.9, 1.5 mM MgCl_2_, 10 mM KCl, 0.5 mM dithiothreitol (DTT) plus protease and phosphatase inhibitor cocktail mix). Nonidet P40 was added to a final concentration of 0.6% and vortexed for 10 s. Nuclei were separated from the cytosolic extracts by centrifugation at 4 °C for 1 min at 1000 × *g*. The nucleus fractions were then washed once with buffer A and then incubated with buffer B (20 mM Hepes pH 7.9, 25% glycerol, 400 nM NaCl, 1 mM ethylenediamine tetraacetic acid (EDTA), 0.5 mM dithiothreitol (DTT) plus protease and phosphatase inhibitor cocktail mix) for 30 min with gentle rocking at 4 °C. The suspensions were centrifugated at 15,000 × *g* for 15 min at 4 °C. Equal quantities of protein were separated on 10% SDS-PAGE gels and were transferred to PVDF membranes (Immobilon-P, Millipore, UK). The blots were probed with anti-phospho-PKC^Thr514^ (1:1000 (0.101 μg/ml); rabbit 9379, Cell Signaling), anti-PKC (1:1000; mouse clone M110 05–983, MERCK), anti-phospho-p44/p42^Thr202/Tyr204^ MAPK (ERK1/2) (1:1000 (0.191 μg/ml); rabbit 9101, Cell Signaling), anti-p44/p42 (ERK1/2) (1:1000 (75 ng/ml); mouse 9107, Cell Signaling), anti-phospho-SAPK/JNK^Thr183/Tyr185^ (1:1000 (0.27 μg/ml); rabbit 4671, Cell Signaling), anti-CD40L (1:700 (0,71 μg/ml); rabbit ab2391, AbCam, Cambridge, UK), anti-Syk (1:1000 (73 ng/ml); rabbit 2712, Cell Signaling) which detects the 72 kDa and the 40 kDa because it is generated using an epitope at the carboxyl terminal, anti-PKCβI/II (1:1000 (1 μg/ml); rabbit SAB4502358, Sigma), anti-PKCγ (1:1000 (0.196 μg/ml); rabbit 43806, Cell Signaling) and anti-βIII tubulin (1:90,000 (5.55 ng/ml); mouse MAB1195, R&D). Binding of the primary antibodies was visualized with HRP-conjugated donkey anti-rabbit or anti-mouse secondary antibodies (1:5000 (0.2 μg/ml); rabbit W4011, mouse W4021, Promega, Southampton, UK) and EZ-ECL kit Enhanced Chemiluminescence Detection Kit (Biological Industries, Geneflow Limited, Staffordshire, UK). Densitometry was carried out using ImageJ software (NIH) of the bands that correspond to the 80 and 82 kDa isoforms detected with anti-phosphoPKC, PKC and PKCβ (I/II), the 44 kDa and 42 kDa isoforms of ERK1 and ERK2, respectively, and the 46 kDa and 54 kDa isoforms of JNK.

## Results

### Phosphorylation of PKC, ERK, and JNK following activation of CD40L reverse signaling

CD40-activated CD40L-mediated reverse signaling is the mechanism involved in the regulation of the growth and elaboration of axons and dendrites in the hippocampus, without the involvement of forward signaling [18]. We used western blotting to assess the degree of phosphorylation and hence activation of PKC, ERK1/ERK2, and JNK in hippocampal pyramidal neuron cultures after CD40L reverse signaling was experimentally activated. To eliminate the complication of endogenous CD40/CD40L signaling confounding the analysis, experiments were carried out on pyramidal neurons cultured from *Cd40*^−/−^ mice because CD40/CD40L signaling is eliminated in these neurons. CD40L reverse signaling was specifically activated by treating these neurons with a chimeric CD40-Fc protein. Fc protein, which does not activate signaling, was used in place of CD40-Fc in control experiments. The chimeric CD40-Fc protein consists of the extracellular domain of CD40 linked to the Fc part of human IgG1 [15, 18]. The role CD40L reverse signaling in promoting dendrite and axon growth from hippocampal pyramidal neurons is based on the previously reported complete rescue of the reduced growth phenotype of cultured *Cd40*^−/−^ neurons with CD40-Fc and the replication of the phenotype of *Cd40*^−/−^ neurons in *Cd40*^+/+^ neurons by treating them with soluble CD40L, which competes with endogenous membrane-integrated CD40L for binding to endogenous CD40 [18]. These latter analyses and experiments on neurons cultured from *Cd40*^+/+^ mice, which would be difficult to interpret, were not repeated here.

*Cd40*^−/−^ neurons were initially grown without CD40-Fc and Fc and were treated for different times with these reagents before lysates were analyzed by western blotting for the levels of particular phospho-proteins after a total of 8 days in vitro. As noted previously [18] and verified here, we did not observe any difference in viability or cell density between neurons cultured from *Cd40*^+/+^ and *Cd40*^−/−^ mice and between neurons cultured from *Cd40*^−/−^ mice treated with either Fc or CD40-Fc. The neurons showed healthy morphologies without any sign of cell death (dead floating detached neurons, dying vacuolated neurons with shrunk soma) in all cultures. Antibodies that recognize phospho-PKC^Thr514^, phospho-ERK1/ERK2^Thr202/Tyr204^, and phospho-JNK^Thr183/Tyr185^ were used. To ensure similar gel loading, anti-βIII tubulin was used as the loading control for the cytosolic fraction (pPKC and pERK1/pERK2) and naphthol blue was used as the loading control for the nuclear fraction (pJNK).

**Fig. 1:**
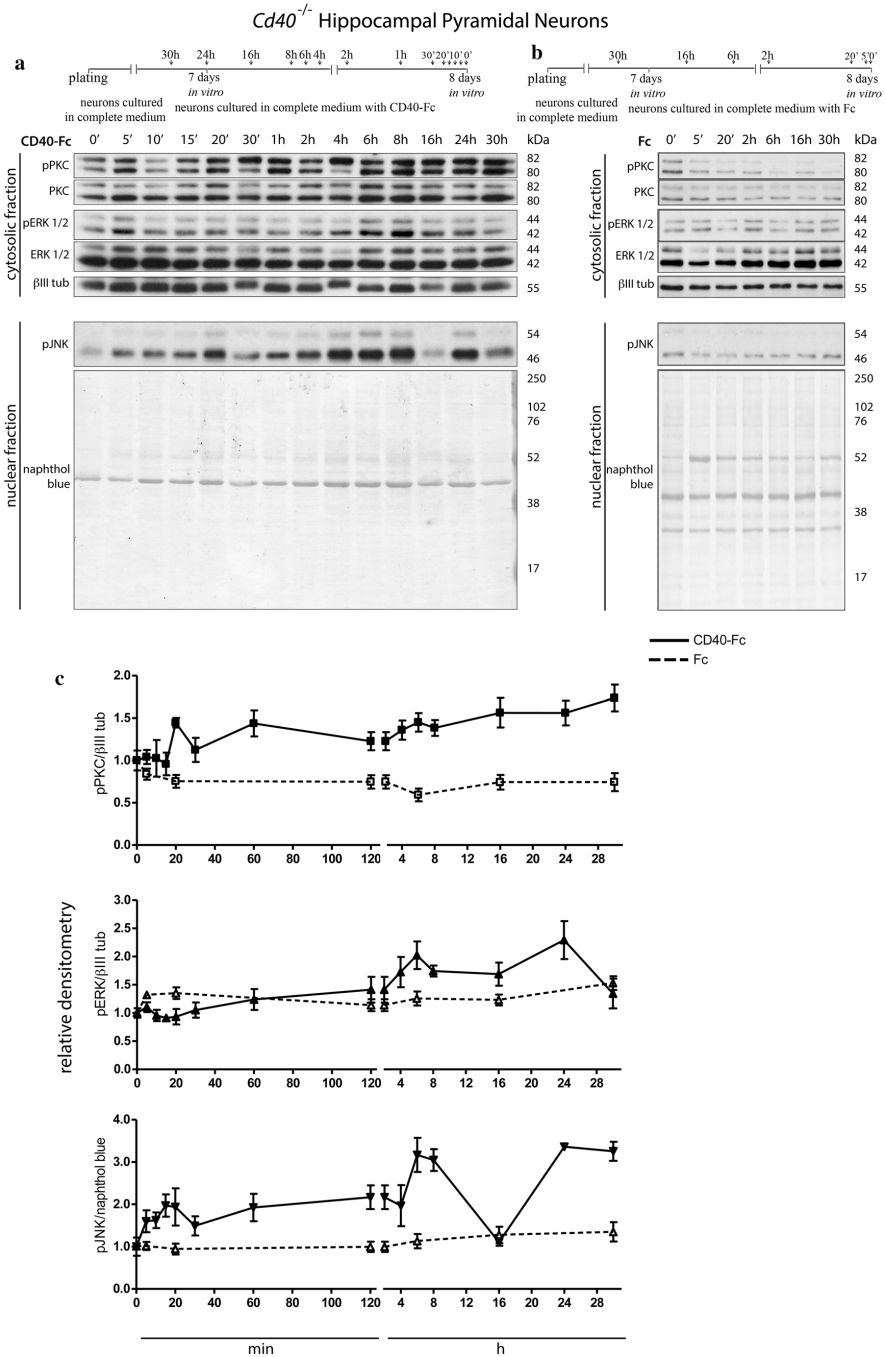
Phosphorylation of PKC, ERK1/ERK2, and JNK after stimulating CD40L reverse signaling. **a**,** b** Schematic illustrations of the experimental protocols and representative Western blots of lysates of *Cd40*^−/−^ E18 hippocampal neuron cultures treated for the indicated times with 1 μg/ml CD40-Fc (**a**) or 1 μg/ml Fc protein as a control (**b**). Lysates were prepared from all cultures after a total of 8 days in vitro. The blots were labeled with anti-phopho-PKC^Thr514^ (pPKC), anti-phospho-p44/p42^Thr202/Tyr204^ MAPK (ERK1/2) (pERK 1/2), anti-phospho-SAPK/JNK^Thr183/Tyr185^ (pJNK). Anti-PKC (PKC), anti-p44/p42 (ERK1/2), and anti-βIII tubulin (βIII tub) were used as loading control for the cytosolic fractions and naphthol blue was used as loading control in the nuclear fraction. **c** Densitometry of at least three independent Western blots (mean ± s.e.m.)

All three phospho-proteins were detectable in unstimulated cultures (0 h) and the levels were unchanged throughout the observation period (30 h) by treatment with Fc control protein (Fig. 1). This suggests a degree of constituent activity in PKC, ERK1/ERK2, and JNK signaling pathways. There were significant increases in the levels of all three phospho-proteins in CD40-Fc-treated cultures compared with Fc-treated cultures, although there were differences in the time course of the increases (Fig. 1c). Whereas there were clear increases in pPKC and pJNK within 20 min, the increase in pERK1/pERK2 started to become evident after 2 h of stimulation. Apart from a consistent and transient return of pJNK levels to baseline at 16 h and a return of pERK1/pERK2 to the baseline after 24 h, the levels of all phospho-proteins remained elevated during the first day. These results suggested that CD40L-mediated reverse signaling increases the phosphorylation and activation in all three signaling pathways.

### Pharmacological manipulation of dendrite and axon growth from cultured hippocampal pyramidal neurons

**Fig. 2:**
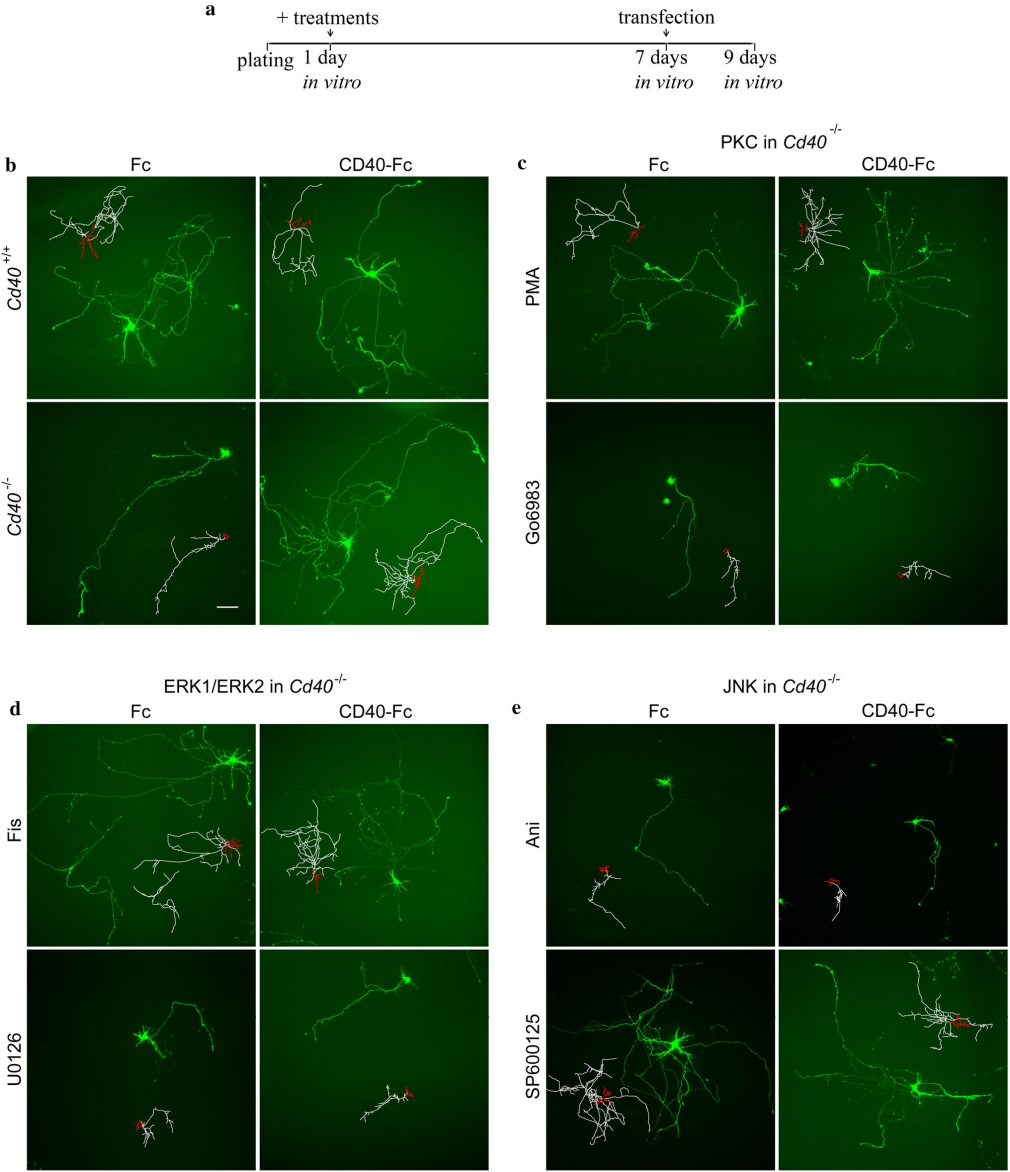
Photomicrographs of Fc-treated and CD40-Fc-treated hippocampal pyramidal neurons exposed to pharmacological reagents. **a** Schematic illustration of the experimental protocol. Treatment reagents were added 24 h after plating. At 7 days in vitro, cultures were transfected with GFP expression vector and were fixed 2 days later. **b**–**e** Cultures of hippocampal neurons were established from E18 *Cd40*^+/+^ (**b**) and *Cd40*^−/−^ (**c**–**e**) embryos. The cultures were treated with either 1 μg/ml of CD40-Fc or 1 μg/ml of control Fc (**b**) together with pharmacological manipulators of PKC (either 500 nM PMA or 500 nM Go6983) (**c**), ERK1/ERK2 (either 1 μM Fis or 1 μM U0126) (**d**), and JNK (either 50 nM Ani or 1 μM SP600125) (**e**). Representative images are shown with scaled sketches of the neuron with the axon in white and dendrites in red. Scale bar, 100 μm

To begin to investigate the significance of PKC, ERK1/ERK2, and JNK in the enhanced dendrite and axon growth response to CD40L reverse signaling, we used specific inhibitors and activators of the PKC, ERK1/ERK2, and JNK signaling pathways. In these experiments, pharmacological reagents plus either CD40-Fc or Fc were added 24 h after plating the neurons which were imaged and analyzed 8 days later (Fig. 2a). While activation of signaling pathways was evident on western blots shortly after stimulation, it takes much longer to observe and quantify the changes produced by these reagents on axon and dendrite growth. Dendrites and axons were clearly distinguishable by their morphology, which permitted their lengths to be separately quantified [13, 25, 26].

We have previously shown that hippocampal pyramidal neurons cultured from *Cd40*^−/−^ embryos replicate the in vivo phenotype of these neurons and that activation of CD40L reverse signaling by treatment with a CD40-Fc chimera recovers the wild-type phenotype [18]. As a starting point, this important observation was replicated and is illustrated in Fig. 2b. Fc-treated hippocampal neurons cultured from *Cd40*^+/+^ embryos exhibited exuberant neurite outgrowth compared with Fc-treated neurons cultured from *Cd40*^−/−^ embryos. Treatment of neurons from *Cd40*^−/−^ embryos with CD40-Fc promoted exuberant neurite similar to that observed in wild-type neurons.

**Fig. 3:**
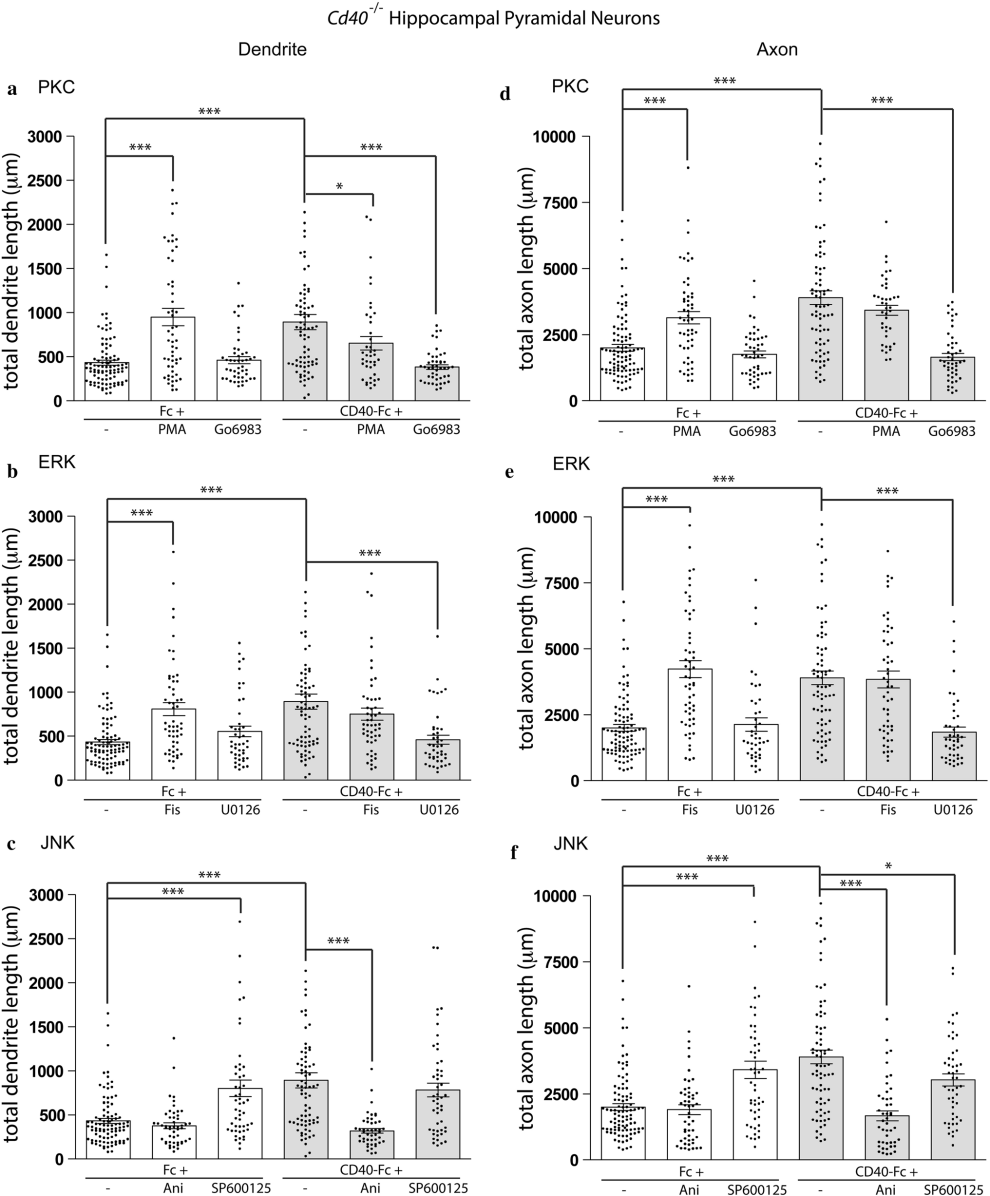
Quantification of the influence of pharmacological reagents on dendrite and axon growth from hippocampal pyramidal neurons. **a**–**f** Scatter charts of total dendrite lengths (**a**–**c**) and axon lengths (**d**–**f**) of hippocampal pyramidal neurons of E18 *Cd40*^−/−^ embryos cultured for 9 days. The neurons were treated 24 h after plating with either 1 μg/ml CD40-Fc (grey bars) or 1 μg/ml control Fc (clear bars) and either received no further treatment or were also treated at this time with the pharmacological reagents indicated: 500 nM PMA, 500 nM Go6983 (**a** and **d**), 1 μM Fis, 1 μM U0126 (**b** and **e**), 50 nM Ani and 1 μM SP600125 (**c** and **f**). The mean ± s.e.m of at least three independent experiments is shown. The dots represent the data obtained from individual neurons (mean of > 60 neurons per condition). One-way ANOVA with multiple Newman–Keuls statistical comparison. Graphs show the statistical significance of the drugs in the presence of Fc (control) or CD40-Fc (when CD40L reverse signaling is activated), and as a reference between Fc and CD40-Fc, ***p < 0.001 and *p < 0.05. The rest of statistical comparisons was as follows: Dendrites: PKC: ***p < 0.001 (PMA vs Go6983) (PMA vs CD40-Fc + Go6983) (Go6983 *vs* CD40-Fc); *p < 0.05 (PMA vs CD40-Fc + PMA). ERK1/2: **p < 0.01 (Fc vs CD40-Fc + Fis) (Fis *vs* CD40-Fc + U0126) (U0126 *vs* CD40-Fc); *p < 0.05 (Fis vs U0126) (CD40-Fc + Fis vs CD40-Fc + U0126). JNK: ***p < 0.001 (Fc vs CD40-Fc + SP600125) (Ani vs SP600125) (Ani vs CD40-Fc) (Ani vs CD40-Fc + SP600125) (SP600125 *vs* CD40-Fc + Ani) (CD40-Fc + Ani *vs* CD40-Fc + SP600125). Axons: PKC: ***p < 0.001 (Fc *vs* CD40-Fc + PMA) (PMA *vs* Go6983) (PMA *vs* CD40-Fc + Go6983) (Go6983 *vs* CD40-Fc) (Go6983 *vs* CD40-Fc + PMA) (CD40-Fc + PMA *vs* CD40-Fc + Go6983); *p < 0.05 (PMA *vs* CD40-Fc). ERK1/2: ***p < 0.001 (Fc *vs* CD40-Fc + Fis) (Fis *vs* U0126) (Fis *vs* CD40-Fc + U0126) (U0126 *vs* CD40-Fc) (U0126 *vs* CD40-Fc + Fis) (CD40-Fc + Fis *vs* CD40-Fc + U0126). JNK: ***p < 0.001 (Fc *vs* CD40-Fc + SP600125) (Ani *vs* SP600125) (Ani *vs* CD40-Fc) (SP600125 *vs* CD40-Fc + Ani); **p < 0.01 (Ani *vs* CD40-Fc + SP600125) (CD40-Fc + Ani *vs* CD40-Fc + SP600125)

Representative images of Fc-treated and CD40-Fc-treated *Cd40*^−/−^ hippocampal pyramidal neurons exposed to different pharmacological reagents are shown in Fig. 2c–e and quantification of dendrite and axon lengths of multiple neurons are shown in Fig. 3. We first confirmed our published findings that the pan-PKC inhibitor Go6983 prevented the in vitro rescue of the phenotype of CD40-deficient neurons with CD40-Fc (Fig. 2c). There were no significant differences in dendrite length (Fig. 3a) and axon length (Fig. 3d) between neurons treated with CD40-Fc plus Go6983 and neurons treated with either Fc protein or Go6983 alone. Mimicking PKC activation with phorbol-12-myristate 13-acetate (PMA), an analogue of diacylglycerol that activates conventional PKCs (PKCα, PKCβ, and PKCγ) and novel PKCs (PKCδ, PKCε, PKCθ, and PKCη) [28], had very similar effects on dendrite and axon growth from CD40-deficient pyramidal neurons as CD40-Fc treatment (Figs. 2c, 3a, d). These observations confirm the importance of PKC activation in mediating the dendrite and axon growth responses of pyramidal neurons to CD40L reverse signaling.

Activation of ERK1/ERK2 was prevented by treating the neurons with U0126, a selective MEK1/MEK2 inhibitor that interferes with MEK1/MEK2-dependent activation of ERK1/ERK2. U0126 prevented rescue of the reduced dendrite growth phenotype of *Cd40*^−/−^ pyramidal neurons by CD40-Fc (Fig. 2d). There were no significant differences in dendrite length (Fig. 3b) and axon length (Fig. 3e) of *Cd40*^−/−^ pyramidal neurons treated with CD40-Fc plus U0126 and neurons treated with Fc protein. There were also no significant changes in dendrite and axon lengths between *Cd40*^−/−^ pyramidal neurons treated with Fc and U0126, showing that U0126 has no effect on dendrite and axon growth alone. U0124, the control inactive U0126 analogue, did not prevent the rescue of the reduced dendrite and axon growth phenotype from *Cd40*^−/−^ pyramidal neurons by CD40-Fc and had no effect on dendrite and axon growth alone (not shown). ERK1/ERK2 was activated by the flavonoid fisetin (Fis) [29, 30]. Fis treatment restored the reduced dendrite and axon growth phenotype of *Cd40*^−/−^ pyramidal neurons as effectively as CD40-Fc and did not significantly affect the extent of dendrite and axon growth from *Cd40*^−/−^ pyramidal neurons treated with CD40-Fc (Figs. 2d, 3b, e). Taken together, these results suggest that ERK1/ERK2 activation contributes to the dendrite and axon growth responses of hippocampal pyramidal neurons to CD40L reverse signaling.

In contrast to the importance of PKC and ERK1/ERK2 activation in enhancing dendrite and axon growth in response of hippocampal neurons to CD40L reverse signaling, pharmacological manipulation of JNK activity suggested that it has the opposite effect on dendrite and axon growth. SP600125, which prevents JNK activation [31], did not prevent CD40-Fc from rescuing the short neurite phenotype of *Cd40*^−/−^ pyramidal neurons (Fig. 2e). There was no significant difference in dendrite length of *Cd40*^−/−^ pyramidal neurons treated CD40-Fc plus SP600125 compared with neurons treated with CD40-Fc alone (Fig. 3c). There was only a small statistically significant reduction in axon length in neurons treated with CD40-Fc plus SP600125 compared with those treated with CD40-Fc alone (Fig. 3f). Moreover, SP600125 promoted dendrite and axon growth as effectively as CD40-Fc (Fig. 3c, f). This suggests the JNK activation is not required for the growth response of pyramidal neurons to CD40L reverse signaling. Accordingly, anisomycin (Ani), a translational inhibitor secreted by Streptomyces which activates JNK [32], did not enhance dendrite and axon growth from pyramidal neurons but prevented CD40-Fc from enhancing dendrite and axon growth (Fig. 3c, f). This suggests that JNK activity is inversely related to the extent of dendrite and axon growth and that it can override the growth-promoting action of CD40L reverse signaling. Taken together, the above findings suggest that PKC and ERK1/ERK2 activity and JNK activity have opposing influences on dendrite and axon growth from pyramidal neurons in response to CD40L reverse signaling.

### The effects of manipulating signaling pathways in combination

**Fig. 4:**
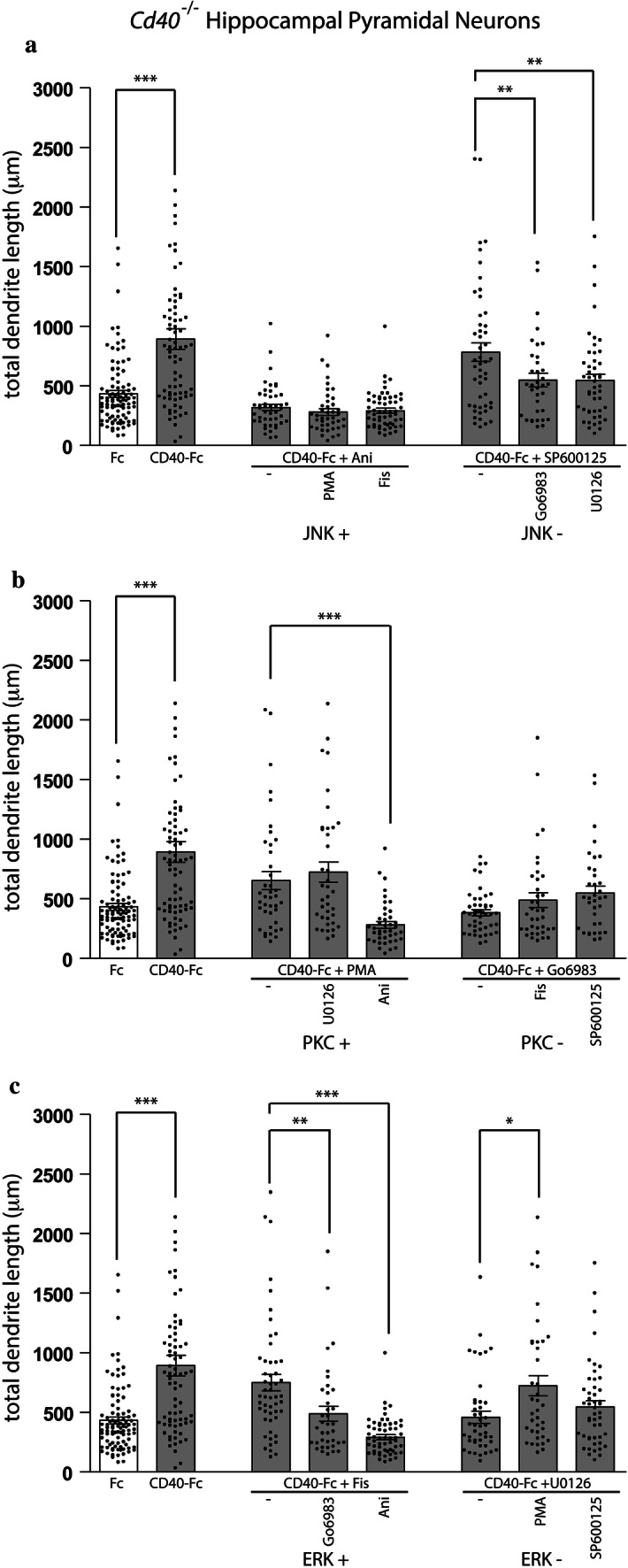
The influence of pharmacological reagents in combination on dendrite growth from hippocampal pyramidal neurons. **a**–**c** Scatter charts of total dendrite lengths of hippocampal pyramidal neurons of E18 *Cd40*^−/−^ embryos cultured for 9 days in vitro and treated 24 h after plating with 1 μg/ml CD40-Fc (grey bars) plus either activators or inhibitors of JNK (**a**), PKC (**b**) or ERK1/ERK2 (**c**) in combination with the activators or/and inhibitors of the other two pathways (the same concentrations were used as in Fig. 3). For comparison, dendrite lengths of neurons in cultures treated with 1 μg/ml control Fc alone (clear bars) are shown. The mean ± s.e.m of at least three independent experiments is shown. The dots represent the data obtained from individual neurons (mean of > 50 neurons per condition). One-way ANOVA with multiple Newman–Keuls statistical comparison, ***p < 0.001, **p < 0.01 and *p < 0.05

**Fig. 5:**
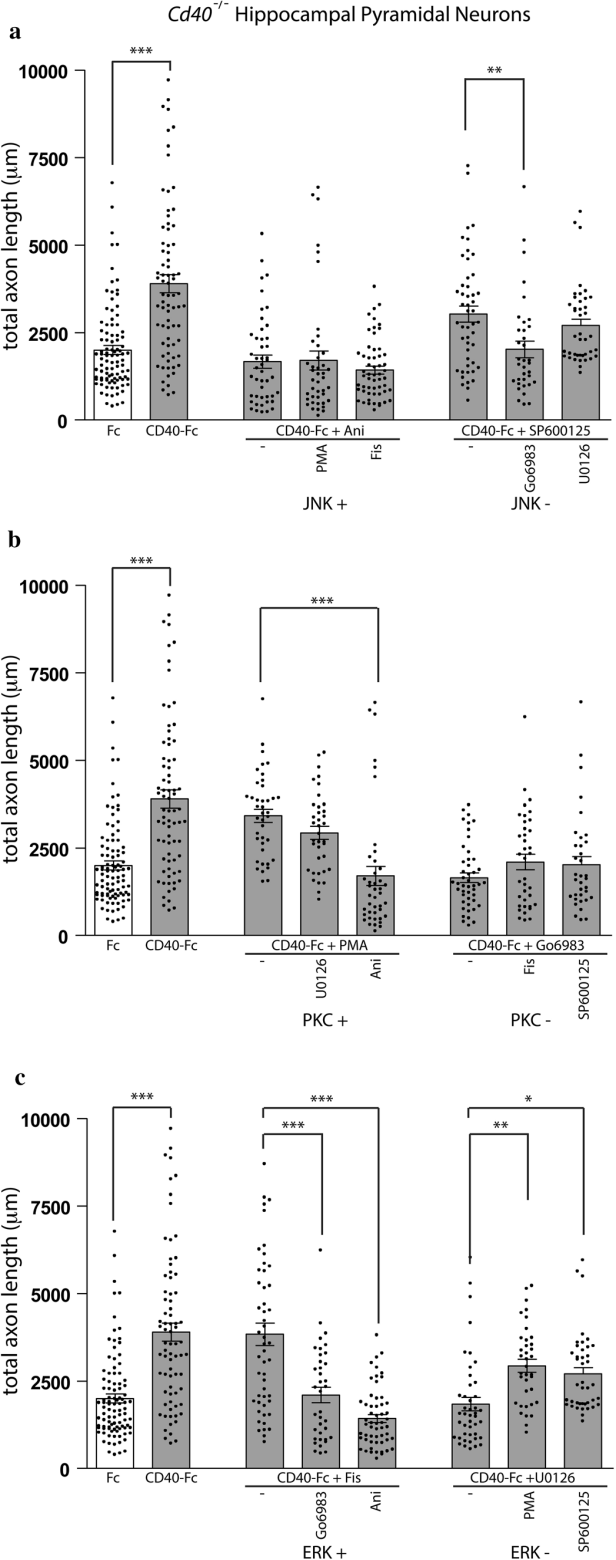
The influence of pharmacological reagents in combination on axon growth from hippocampal pyramidal neurons. **a**–**c** Scatter charts of total axons lengths of hippocampal pyramidal neurons of E18 *Cd40*^−/−^ embryos cultured for 9 days in vitro and treated 24 h after plating with 1 μg/ml CD40-Fc (grey bars) plus either activators or inhibitors of JNK (**a**), PKC (**b**) or ERK1/ERK2 (**c**) in combination with the activators or/and inhibitors of the other two pathways (the same concentrations were used as in Fig. 3). For comparison, axon lengths of neurons in cultures treated with 1 μg/ml control Fc alone (clear bars) are shown. The mean ± s.e.m of at least three independent experiments is shown. The dots represent the data obtained from individual neurons (mean of > 50 neurons per condition). One-way ANOVA with multiple Newman-Keuls statistical comparison, ***p < 0.001, **p < 0.01 and *p < 0.05

In the experiments of the last section, we studied the influence of PKC, ERK, and JNK in regulating the growth response of pyramidal neurons to CD40-activated CD40L reverse signaling. To gain an understanding of the relative importance of PKC, ERK and JNK, and their potential functional interactions in regulating the growth response of pyramidal neurons to CD40-activated CD40L reverse signaling, we studied the effects of pharmacological reagents in combination. To simplify these experiments, we treated *Cd40*^−/−^ pyramidal neurons with CD40-Fc and either activated or inhibited JNK, PKC, and ERK1/ERK2 either alone or together with a pharmacological reagent that manipulates each of the other pathways in a way that has the opposite effect on growth. For example, CD40-Fc-stimulated neurons were treated with Ani (which activates JNK and inhibits the CD40-Fc growth response) either alone or in combination with either PMA or Fis (which promote growth by activating PKC and ERK1/ERK2, respectively). The protocol for this experiment was the same as for the individual treatments, i.e., neuronal cultures were treated 24 h after plating with the combined reagents and analyzed after a total of 9 days in vitro. For comparison, we measured dendrite and axon growth from neurons treated with either CD40-Fc or Fc alone. Quantification of dendrite length and axon length in these experiments is shown in Figs. 4 and 5, respectively.

The extent of dendrite and axon growth from pyramidal neurons treated with Ani, which eliminated CD40-Fc-promoted dendrite and axon growth (Figs. 3, 4a and 5a), was unaffected by concomitant treatment of these neurons with either PMA or Fis, which otherwise promoted dendrite and axon growth as effectively as CD40-Fc alone (Fig. 3). Figures 4a and 5a show that there were no significant differences in CD40-Fc-promoted dendrite and axon length in cultures treated with Ani and cultures treated with either Ani plus PMA or Ani plus Fis. This suggests that the growth inhibitory effect of JNK activation on CD40-Fc-promoted dendrite and axon growth is dominant to the growth-promoting effect of PKC and ERK1/ERK2 activation.

The inactivation of JNK with SP600125, which promoted dendrite and axon growth as effectively as CD40-Fc and did not interfere with CD40-Fc-promoted growth (Figs. 3, 4a and 5a), was partially reduced by concomitant treatment with either Go6083 or U0126, that otherwise completely eliminated CD40-Fc-promoted dendrite and axon growth (Fig. 3). Interestingly, there were differences in the responses to dendrites and axons to combined reagents. Whereas there were significant reductions in dendrite length in cultures treated with SP600125 plus Go6083 and SP600125 plus U0126 compared with cultures treated with SP600125 alone (Fig. 4a), a significant reduction in axon length was only observed in cultures treated with SP600125 plus Go6083 compared with cultures treated with SP600125 alone (Fig. 5a). This suggests that PKC activity and ERK1/ERK2 activity are able to modulate dendrite growth when JNK activity is reduced and that only PKC activity is able to modulate axon growth when JNK activity is reduced.

The activation of PKC with PMA, which promoted dendrite and axon growth as effectively as CD40-Fc (Figs. 3, 4b and 5b), was unaffected by concomitant treatment with U0126 which otherwise eliminates CD40-Fc-promoted growth (Fig. 3). Figures 4b and 5b show that there were no significant differences in CD40-Fc-promoted dendrite and axon length in cultures treated with PMA and cultures treated with either PMA plus U0126. Accordingly, the inactivation of PKC with Go6083, which completely eliminated CD40-Fc-promoted dendrite and axon growth (Figs. 3, 4b and 5b), was unaffected by the concomitant presence of Fis which otherwise promoted dendrite and axon growth as effectively as CD40-Fc (Fig. 3). Figures 4b and 5b show that there were no significant differences in dendrite and axon length in cultures treated with Fis plus Go6083 compared with cultures treated with Go6083 alone. Taken together, these observations suggest that the influence of PKC activity on dendrite and axon growth is dominant to ERK1/ERK2 activation.

In agreement with the predominant role of JNK and PKC over ERK1/ERK2 in CD40-Fc promoted growth, the dendrite and axon length observed when ERK1/ERK2 pathway was manipulated was altered when we manipulated the other two pathways. Activation of ERK1/ERK2 with Fis, which promoted dendrite and axon growth as effectively as CD40-Fc (Figs. 3, 4c and 5c), was significantly reduced by concomitant treatment with Go6083 or Ani, that otherwise eliminate CD40-Fc-promoted growth (Fig. 3). Figures 4c and 5c show that there were significant differences in CD40-Fc-promoted dendrite and axon length in cultures treated with Fis and cultures treated with either Fis plus Go6983 or Fis plus Ani. The complete elimination CD40-Fc-promoted dendrite and axon growth by inactivation of ERK1/ERK2 with U0126 (Figs. 3, 4c and 5c) was partially prevented by concomitant treatment with PMA and SP600125. As shown above, there was a difference between the responses of dendrites and axons to combinations of pharmacological reagents SP600125 and U0126. Whereas PMA caused a significant reversal of the inhibition of CD40-Fc-induced dendrite growth by U0126 (Fig. 4c), both PMA and SP600125 each caused significant reversals of the inhibition of CD40-Fc-induced axon growth by U0126 (Fig. 5c).

In summary, these results indicate that inactivation of JNK is required for the dendrite and axon growth responses of pyramidal neurons to CD40-Fc and that activation of JNK is able to abolish the CD40-Fc growth responses. Moreover, the effect of JNK activity on dendrite and axon growth overrides that of PKC and ERK activity. Whereas, enhanced PKC activity and enhanced ERK activity increase growth, the influence of ERK activity on growth is modulated by PKC activity. These results imply that JNK, PKC, and ERK participate in an interacting network that regulates growth.

### Activity of signaling pathways following pharmacological manipulation

**Fig. 6:**
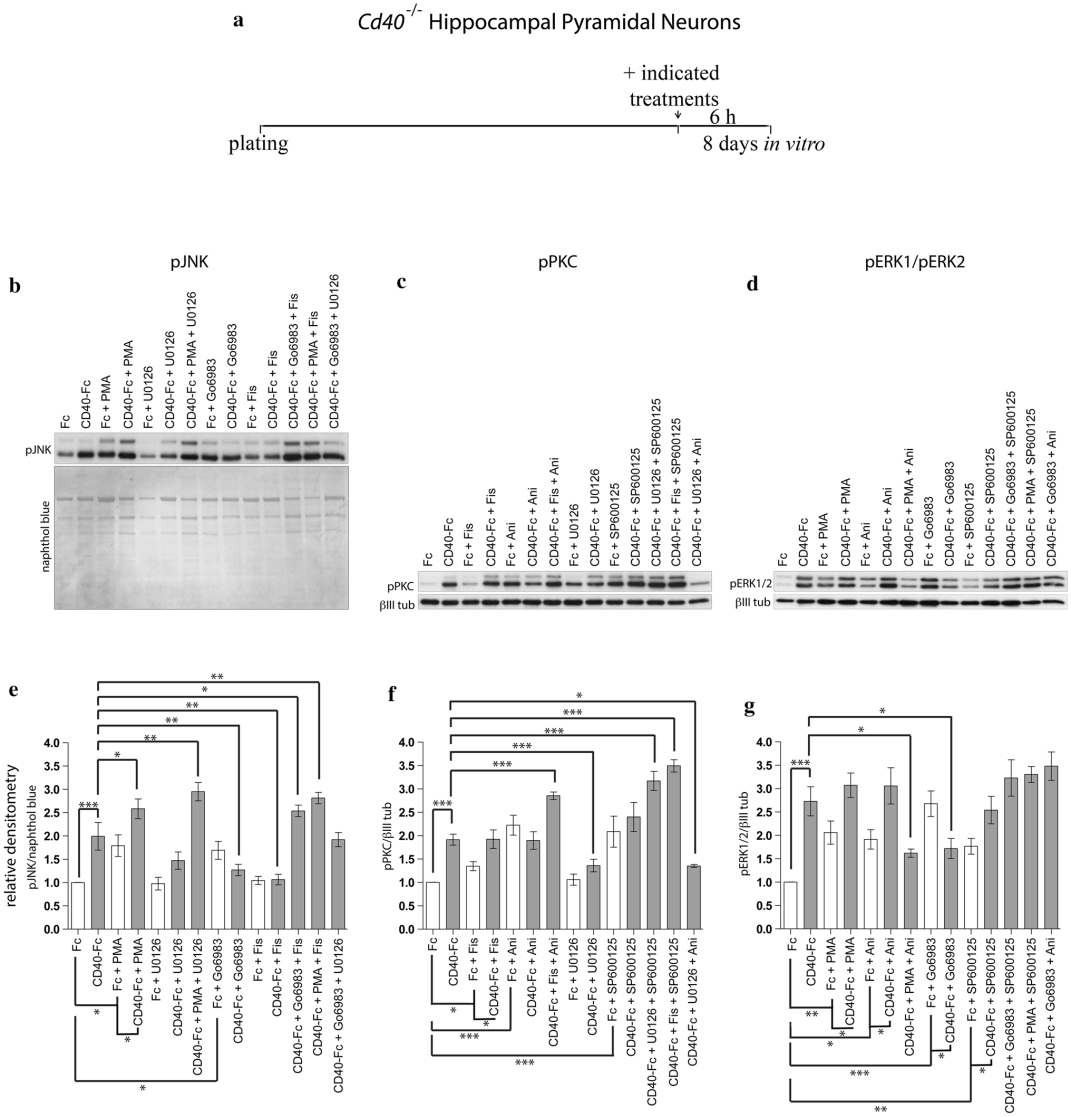
Regulation of JNK, PKC, and ERK1/ERK2 phosphorylation by pharmacological reagents in hippocampal neurons in the presence and absence of CD40L reverse signaling. **a** Schematic illustration of the experimental protocol. The neurons were treated 8 days after plating with either 1 μg/ml CD40-Fc or 1 μg/ml control Fc in combination with the reagents indicated for 6 h. The concentrations were the same as those indicated in Fig. 3. **b**–**d** Representative western blots of lysates of hippocampal neuron of *Cd40*^−/−^ E18 embryos. The western blots were probed with anti-pJNK after treatment with activators and inhibitors of PKC and ERK1/ERK2 (**b**), anti-pPKC after treatment with activators and inhibitors of ERK1/ERK2 and JNK (**c**) and pERK1/ERK2 after treatment with activators and inhibitors of PKC and JNK (**d**). Anti-βIII tubulin was used to normalize western blots of cytosolic fractions and Naphthol blue for the nuclear fractions. **e**–**g** Quantification of at least three independent western blots. The grey bars show combined treatments in the presence of 1 μg/ml CD40-Fc and the clear bars show combined treatments in the presence of 1 μg/ml Fc. The mean ± s.e.m is indicated (***p < 0.001, **p < 0.01 and *p < 0.05, one-way ANOVA with multiple Newman-Keuls statistical comparison). Statistical not presented in the graphs were as follows: **e**, pJNK ***p < 0.001 (CD40-Fc + U0126 vs CD40-Fc + PMA + U0126) (CD40-Fc + Fis vs CD40-Fc + PMA + Fis) (CD40-Fc + Fis vs CD40-Fc + Go6983 + Fis) (CD40-Fc + Go6983 vs CD40-Fc + Go6983 + Fis); **f**, pPKC ***p < 0.001 (CD40-Fc + Fis vs CD40-Fc + Fis + Ani) (CD40-Fc + Ani vs CD40-Fc + Fis + Ani) (CD40-Fc + U0126 vs CD40-Fc + U0126 + SP600125) (CD40-Fc + Fis vs CD40-Fc + Fis + SP600125) **p < 0.01 (CD40-Fc + SP600125 vs CD40-Fc + U0126 + SP600125) *p < 0.05 (CD40-Fc + SP600125 vs CD40-Fc + Fis + SP600125); **g**, pERK1/2 **p < 0.01 (CD40-Fc + PMA vs CD40-Fc + PMA + Ani) (CD40-Fc + Ani vs CD40-Fc + PMA + Ani) (CD40-Fc + Go6983 vs CD40-Fc + Go6983 + Ani) *p < 0.05 (CD40-Fc + Go6983 vs CD40-Fc + Go6983 + SP600125)

We used western blotting to access the degree of phosphorylation and hence activity of JNK, PKC, and ERK1/ERK2 following pharmacological manipulation of these signaling pathways. Neurons were plated with complete medium and 8 days later, neurons were treated for 6 h with either Fc or CD40-Fc with and without pharmacological reagents that either inhibit or activate different pathways singularly and in combination (Fig. 6a). After 6 h, when all three phospho-proteins were clearly increased by CD40-Fc treatment (Fig. 1), lysates were probed for either phospho-JNK, phospho-PKC or phospho-ERK1/phospho-ERK2. The aim of these studies was to determine the extent to which pharmacological manipulation of either one signaling pathway or two pathways in combination affected activity of the other pathway in the presence and absence of CD40-Fc. Figure 6b–d show representative western blots for pJNK, pPKC, and pERK1/pERK2, respectively, and Fig. 6e–g summary the densitometry from multiple blots.

There were highly significant increases in pJNK, pPKC, and pERK1/pERK2 in CD40-Fc-treated cultures compared with Fc-treated cultures, confirming that CD40L reverse signaling enhanced phosphorylation of each signaling protein by 6 h (Fig. 6e–g). In the absence of CD40L reverse signaling, the level of pJNK was significantly increased by either activation or inactivation of PKC (Fig. 6e). In the presence of CD40L reverse signaling, activation of PKC also significantly increased the level of pJNK relative to its level in cells treated with CD40-Fc alone, but inactivation of PKC significantly decreased the level of pJNK (Fig. 6e). In the absence of CD40L reverse signaling, the level of pJNK was not significantly affected by either activation or inactivation of ERK1/ERK2 (Fig. 6e). In the presence of CD40L reverse signaling, activation of ERK1/ERK2 significantly decreased the level of pJNK. While activation of PKC alone significantly increased the level of pJNK, PMA in combination with either Fis or U0126, that alone produce low levels of pJNK, caused further significant increases in the level of pJNK. Interestingly, although activation of ERK1/ERK2 and inactivation of PKC each caused small, significant decreases in the level of pJNK, when Fis and Go6983 were used in combination, there was a significant increase in the level of pJNK in CD40-Fc-treated neurons (Fig. 6e). These findings show that when CD40L reverse signaling is activated, the level of pJNK is regulated by both PKC and ERK. Furthermore, manipulation of PKC and ERK activity simultaneously (see CD40-Fc + Go6983 + Fis) had distinctive effects on pJNK levels in the presence of CD40L compared with the individual treatments (CD40-Fc + Go6983 and CD40-Fc + Fis). Remarkably, in all combinations in which PKC was activated, independently of the activation state of ERK1/ERK2, the levels of pJNK were elevated.

In the absence of CD40L reverse signaling, the level of pPKC was significantly increased by either activation or inactivation of JNK (Fig. 6f). However, in the presence of CD40L reverse signaling, neither activation nor inactivation of JNK activation significantly affected the level of pPKC (Fig. 6f). In the absence of CD40L reverse signaling, the level of pPKC was significantly increased by ERK1/ERK2 activation, but not significantly affected by ERK1/ERK2 inactivation. However, in the presence of CD40L reverse signaling, ERK1/ERK2 activation had no significant effect on the level of pPKC and ERK1/ERK2 inactivation significantly decreased the level of pPKC. When the activity of ERK1/ERK2 and JNK was manipulated simultaneously in the presence of CD40L reverse signaling, somewhat greater and distinctive effects on the level of pPKC were observed compared with the individual treatments. For example, JNK inactivation together with either ERK1/ERK2 inactivation or ERK1/ERK2 activation caused highly significant increases the level of pPKC (Fig. 6f). The above results show that when CD40L reverse signaling is activated, the level of pPKC is regulated by both JNK and ERK in distinctive ways.

In the absence of CD40L reverse signaling, the level of pERK1/pERK2 was significantly increased by all pharmacological manipulations of JNK and PKC: activation and inactivation of JNK and activation and inactivation of PKC (Fig. 6g). A significant effect on the levels of pERK was observed when CD40L reverse signaling was activated compared with the absence of this activation. However, in the presence of CD40L reverse signaling, significant changes in the levels of pERK were observed with only two pharmacological manipulations of JNK and PKC: both PKC inhibition and PKC activation together with JNK activation caused significant reductions in the level of pERK compared with CD40-Fc treatment (Fig. 6g). The above results show that the level of pERK is regulated by both JNK and PKC, although not as extensively in the presence of CD40L reverse signaling. Overall, with the presence of CD40-Fc, the regulation of pERK levels by JNK and PKC was not as great as the regulation of pJNK by PKC and ERK and the regulation of pPKC by JNK and ERK.

Taken together, the above results suggest that when CD40L was activated, JNK, PKC, and ERK influence the activity of one another and function as regulatory network, with distinctive dynamics. These findings suggest that JNK, PKC, and ERK signaling pathways regulate neural process growth by acting as an interacting network rather than acting in simple linear sequence.

### The Syk tyrosine kinase as a candidate to be a link between CD40L and PKC

To provide a more detailed understanding of how the CD40L reverse signaling receptor links to the intracellular signaling network, we attempted to identify membrane-associated molecular components of the activated CD40L receptor complex. Previous work using specific siRNAs to different PKC isoforms has shown that PKCβ is the main isoform that mediates the neurite growth-promoting effects of CD40L reverse signaling in hippocampal pyramidal neurons [18]. Because PKC activation involves translocation of a latent form from the cytosol to the membrane [33], we initially used an in silico approach to determine possible protein–protein interactions (PPI) between CD40L and PKCβ using the STRING database (https://string-db.org/). Mouse CD40L and PKCβ were analyzed for all possible interactions (textmining, experiments, databases, co-expression, neighborhood, gene fusion and co-occurrence) with reliability scores more than 0.4 (i.e., medium confidence and greater). Supplementary Table 1 lists all annotated PPI with reliability scores of 0.4 and greater for CD40L and PKCβ separately and in common. Supplementary Table 2 shows the most relevant PPIs in common that might be involved in CD40L reverse signaling. These proteins not only include ERK1, ERK2, JNK1, JNK2, JNK3 and several ERK and JNK regulatory proteins, but also spleen tyrosine kinase (Syk). Syk is a non-receptor tyrosine kinase that is most highly expressed in cells of the immune system. It is recruited to the cell membrane following activation of receptors of the adaptive immune response and plays a key role in signal transduction from the receptor complex [34]. In addition, recent work on Syk has been shown to mediate a variety of diverse biological functions in several different cell types [35]. Full-length Syk is a 72 kDa protein that is proteolytically cleaved to generate a 40 kDa fragment that contains the catalytic domain [36].

We used western blotting to analyze the expression of Syk in hippocampal lysates from *Cd40*^+/+^ and *Cd40*^−/−^ mice over a range of ages. Both 40 kDa and 72 kDa components were detectable and displayed a similar pattern of expression in the hippocampi of *Cd40*^+/+^ and *Cd40*^−/−^ mice (Fig. S1). The 40 kDa catalytic fragment was clearly expressed during the perinatal period up to P6, after which there was a clear decrease in expression with age (Fig. S1a). The expression of the 72 kDa full-length protein became evident at P3, and its level increased with age (Fig. S1a). Syk was also detectable in the lysates of hippocampal neuron cultures and the 40 kDa and 72 kDa also showed a reciprocal pattern of expression with time in culture. Whereas the 40 kDa fragment was predominant up to 5 days in vitro, the full-length protein was predominant since 5–7 days in vitro (Fig. S1c).

We also analyzed the expression of PKCβ, PKCγ, and CD40L in hippocampal culture lysates. No remarkable differences were observed in hippocampal lysates between *Cd40*^+/+^ and *Cd40*^−/−^ in the expression of PKCβ (βI/II) and PKCγ (Fig. S1a). However, the expression of CD40L showed some differences between *Cd40*^+/+^ and *Cd40*^−/−^. In CD40-null animals, the expression of CD40L started at postnatal stages and displayed a peak between P3 and P6, while in wild-type mice, the expression began earlier and seemed more sustained within time with a peak in perinatal neurons (Fig. S1a). This pattern of CD40L expression was also observed in neurons cultured from *Cd40*^−/−^ compared with neurons from *Cd40*^+/+^ (Fig. S1b). Comparable levels of expression of PKCβ and PKCγ were observed in hippocampal neurons (Fig. S1c).

### CD40-activated CD40L reverse signaling recruits Syk and PKCβ

**Fig. 7:**
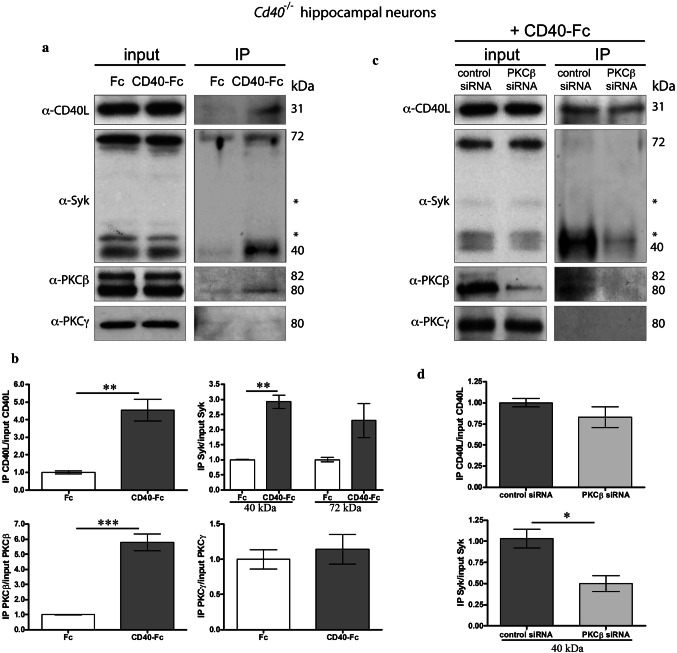
Pull down of Syk and PKCβ in *Cd40*^−/−^ hippocampal neurons after CD40-activated CD40L reverse signaling. **a** Representative western blots of the expression of CD40L, Syk, PKCβ and PKCγ in *Cd40*^−/−^ neurons from E18 embryos cultured for 8 days and treated for 30 min with either 1 μg/ml Fc or 1 μg/ml CD40-Fc, before pulled down Fc fragment (input) and after pulled down Fc fragment (IP). **b** Quantification of three independent experiments of the quantity of CD40L, 40 kDa and 72 kDa Syk, PKCβ and PKCγ after IP normalizing with the total quantity of those proteins in the input. **c** Representative western blots of the expression of CD40L, Syk, PKCβ, and PKCγ in *Cd40*^−/−^ neurons transfected with control siRNA or PKCβ siRNA and treated for 30 min with 1 μg/ml CD40-Fc, before pulled down Fc fragment (input) and after pulled down Fc fragment (IP). **d** Quantification of three independent experiments of the quantity of CD40L and 40 kDa Syk after IP normalizing with the total quantity of those proteins in the input. **b** and** d** ***p < 0.001, **p < 0.01 and *p < 0.05 *t* test. * = non-specific band

While STRING predicted interaction between CD40L, Syk and PKCβ, we conducted immunoprecipitation experiments to determine if these proteins physically interact in culture following activation of CD40L reverse signaling by CD40-Fc. In these experiments, we cultured E18 hippocampal pyramidal neurons from *Cd40*^−/−^ mice for 8 days. These cultures were treated with either CD40-Fc or Fc 30 min before extraction of lysates. After Fc fragments were pulled down in the lysates with protein G-Sepharose, we used western blotting to detect the presence of CD40L, Syk, PKCβ, and PKCγ (Fig. 7a). Quantification of associated proteins after IP is shown in Fig. 7b. As expected, we only detected the association of CD40L in samples treated with CD40-Fc but not in samples treated with Fc (Fig. 7a, b). We detected some unspecific interaction of full-length Syk in samples treated with Fc, but treatment with CD40-Fc increased its presence compared with Fc (Fig. 7a); however, this increase was not statistical significant (Fig. 7b). There was clear detection of the 40 kDa Syk catalytic fragment (Fig. 7a) with statistical significance between neurons treated with CD40-Fc compared with neurons treated with Fc (Fig. 7b). When we analyzed the G-Sepharose fraction for the two main PKCs involved in mediating the grow of processes in pyramidal neurons (PKCβ) and medium spiny neurons (PKCγ) [18], we only detected PKCβ, but not PKCγ, when CD40 reverse signaling was activated with CD40-Fc in *Cd40*^−/−^ hippocampal neurons (Fig. 7a, b). Both PKC isoforms were clear detectable in the lysates prior to G-Sepharose pull down (Fig. 7a).

To analyze the involvement of PKCβ in these interactions, we repeated the above experiments with prior PKCβ down-regulation. To down-regulate PKCβ, we used a specific siRNA and a scrambled siRNA control, as previously reported [18]. In these experiments, we observed clear reduction in the quantity of the 40 kDa Syk fragment (Fig. 7c, d) and did not detect any interaction with full-length Syk (Fig. 7c). It is unclear why in this set of experiments we did not detect the full-length protein. Because the antibody recognizes an epitope at the carboxyl terminal, the lack of detection of full-length protein raises the possibility that Syk may have undergone proteolytic degradation after stimulation with CD40-Fc generating the 40 kDa fragment. No differences were observed in the quantity of CD40L pulled down when PKCβ was down-regulated (Fig. 7c, d). Taken together, the above results suggest that activation of CD40L reverse signaling in hippocampal pyramidal neurons promotes the recruitment of Syk and PKCβ, but not PKCγ, to CD40L and that PKCβ is involved in the recruitment of Syk to this complex.

## Discussion

Given the extensive physiological importance of CD40L reverse signaling in regulating the growth and elaboration of neural processes in the developing nervous system, the aim of the current study was to extend our understanding of the intracellular signaling events that mediate the effect of CD40L reverse signaling on axon and dendrite growth, focusing in on the well-characterized hippocampal pyramidal neuron model. Moreover, by analyzing the participation of multiple signaling pathways in a given neuronal model that have been individually implicated in influencing axon and dendrite growth in a variety of neuronal models, we hoped to show how their function might be integrated in mediating growth responses to a particular physiologically important ligand.

Using a variety of specific pharmacological reagents, we demonstrated that proteins of three ubiquitous signaling pathways that have each been implicated in the regulation of axon and/or dendrite growth in several in vitro neuronal models all play a role in regulating the axon and dendrite growth responses of pyramidal neurons to CD40L reverse signaling. Either activators or inhibitors of JNK, PKC, and ERK1/ERK2 significantly affected in different ways the axon and dendrite growth responses of pyramidal neurons to CD40L reverse signaling. While western blotting showed that CD40L reverse signaling caused an increase in the phosphorylation (and hence the activity) of each of these proteins, experiments using pharmacological reagents suggested that activity was not correlated with enhanced growth for each protein. Inhibitors of PKC and ERK1/ERK2 each eliminated CD40L-promoted dendrite and axon growth, and activators of these signaling proteins generally did not significantly affect CD40L-promoted growth (there was a small statistically reduction of CD40-Fc-promoted dendrite growth by PKC activation). Accordingly, activation of either PKC or ERK1/ERK2 in the absence CD40L reverse signaling promoted the same degree of axon and dendrite growth as CD40L reverse signaling itself. However, JNK activation eliminated the dendrite and axon growth response to CD40L reverse signaling and JNK inactivation generally did not significantly affect the growth response to CD40L reverse signaling (there was a small statistically reduction of CD40-Fc-promoted axon growth by JNK inactivation). Accordingly, JNK inactivation in the absence CD40L reverse signaling promoted the same degree of axon and dendrite growth as CD40L reverse signaling itself. Taken together, these data suggest that, whereas PKC activation and ERK1/ERK2 activation play a role in CD40L-promoted growth, JNK inactivation is important for CD40L-promoted growth. Apart from the minor differences highlighted above, our findings suggest that these proteins play similar roles in axon growth and in dendrite growth from hippocampal pyramidal neurons.

Experiments using combinations of pharmacological reagents revealed the relative importance of PKC, ERK1/ERK2, and JNK and their potential functional interactions in mediating the CD40L reverse signaling growth response. The demonstration that the elimination of the CD40-Fc-promoted growth by JNK activation could not be prevented by concomitant activation of either PKC or ERK suggests that JNK inactivation is necessary for CD40-Fc-promoted growth and plays a more dominant role than PKC and ERK1/ERK2 activation. However, our finding that in the presence of JNK inactivation, inactivation of either PKC or ERK1/ERK2 significantly reduced, but did not eliminate, the CD40-Fc dendrite growth response suggests that PKC or ERK1/ERK2 is able to exert some influence on the CD40-Fc growth response when JNK is reduced. Our demonstration that the elimination of the CD40-Fc growth response by PKC inactivation could not be prevented by ERK/ERK2 activation and that ERK1/ERK2 inactivation did not eliminate the CD40-Fc growth response in neurons in which PKC was activated suggests that PKC exerts a more dominant role than ERK1/ERK2 in the axon and dendrite growth responses of pyramidal neurons to CD40 reverse signaling. Putting all these observations together suggests that JNK, PKC, and ERK1/ERK2 do not mediate the growth response of pyramidal neurons to CD40L reverse signaling by functioning in a simple linear sequence. Rather, they must functionally interact to regulate the growth response.

**Fig. 8:**
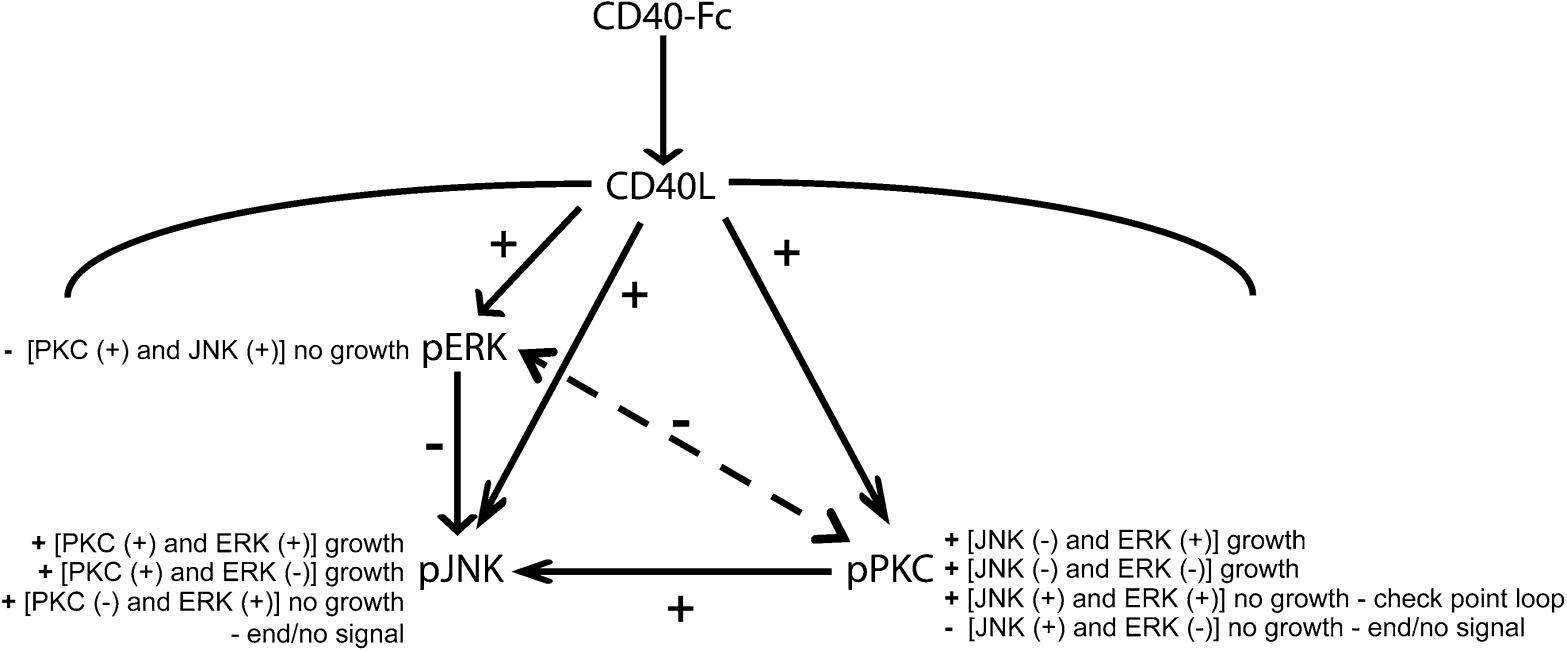
Schematic summary of the results. Graphic summary of the interactions between the PKC, ERK1/ERK2, and JNK downstream of the activation of CD40L reverse signaling. The direction of influence is indicated by the arrows (solid arrows indicate the influence of activation of these pathways and dashed arrows indicate the influence of inhibition of these pathways), change in the level of phosphorylation (+ = increased phosphorylation and – = decreased phosphorylation) and the effect on neural process growth are documented. Activation of CD40L reverse signaling enhances the phosphorylation of JNK, PKC, and ERK1/ERK2. Phosphorylation of JNK is positively modulated by PKC and negatively modulated by ERK1/ERK2, while phosphorylation of PKC and ERK is reciprocally regulated (inhibition of ERK1/ERK2 reduces pPKC and inhibition of PKC reduces pERK1/pERK2). Activation of PKC and JNK reduce the phosphorylation of ERK1/ERK2, which enhances JNK phosphorylation with the concomitant restraint of growth. PKC activation promotes JNK phosphorylation independently of the ERK1/ERK2 status, with a positive net effect on growth compared to Fc, suggesting that the increase in the phosphorylation of JNK may lead to growth termination. However, the inhibition of PKC inhibits CD40L reverse signaling-promoted growth, in both situations, when the inhibition of PKC is together with the activation of ERK1/ERK2 that increases the level of pJNK and when is together with the inhibition of ERK1/ERK2 that does not have any effect on the level of pJNK. The level of phospho-PKC is modulated by activation or inactivation of JNK together with activation or inactivation ERK. However, increased growth was only observed when JNK was inactivated. This raises the possibility that the level of JNK activation may be a checkpoint loop that governs whether growth continues or is terminated depending on the status of PKC and ERK

Our study of the phosphorylation, and hence activation, of JNK, PKC, and ERK1/ERK2 in cultures treated with pharmacological reagents has given us further insight into the regulation of intracellular signaling pathways downstream of CD40L reverse signaling. While the above functional studies have demonstrated that JNK activity has a powerful, dominant inhibitory effect on the growth of neural processes, our phosphorylation studies clearly show that JNK activity is influenced by PKC and ERK1/ERK2 activity, both alone and in combination. Interestingly, when the activity of PKC and ERK1/ERK2 was individually manipulated, only inhibition of ERK1/ERK2 reduced the phosphorylation of PKC and only inhibition of PKC reduced the phosphorylation of ERK1/ERK2. When we manipulated the two protein pathways, in a few instances, when the activity of one signaling pathway is activated or inactivated, positive or negative manipulation of a second pathway has the same effect on the phosphorylation level of the third. For example, when ERK1/ERK2 is activated, either activation or inactivation of PKC increases the level of pJNK, and when the activity of JNK is inactivated either activation or inactivation of ERK1/ERK2 increases the level of pPKC. These observations not only illustrate the interactions between two signaling pathways in influencing the third but also suggest that a fine balance in level of activation of particular pathways influences the activity of the third. The potential interactions between JNK, PKC, and ERK1/ERK2 and their consequences on growing of the neuronal processes are summarized in Fig. 8. Supplemental Fig. 2 complements Fig. 8, showing the effect of all combinations of activators and inhibitors on dendrite and axon growth.

The control of neuronal process growth evolves over time, with several factors participating at different times. Temporal aspects of the interactions between JNK, PKC, and ERK1/ERK2 have not been addressed here, as the very extensive experimental work required to explore this definitely is beyond the scope of the current study. Nonetheless, we have shown that in the absence of pharmacological reagents, the time course of enhanced JNK, PKC, and ERK1/ERK2 phosphorylation following activation of CD40L reverse signaling differs for these three proteins. Perhaps activation of each protein contributes to different aspects of the growth response or even termination of this response. In this respect, it is curious that CD40L reverse signaling increases activity in all three pathways and yet increased JNK activity has a clear inhibitory effect on growth, whereas PKC and ERK1/ERK2 enhance growth. Perhaps the physiological significance of enhanced JNK activation may be related to the control of the termination of the growth response.

Another important aspect of our study was to address how intracellular signaling is initiated downstream of CD40L by determining and analyzing components of the potential CD40L receptor complex. In silico analysis initially suggested that the Syk tyrosine kinase associates with CD40L and PKCβ, the PKC isoform that has shown to mediate the effects of CD40L reverse signaling on the growth of neural processes from hippocampal pyramidal neurons [18]. Immunoprecipitation studies subsequently suggested that Syk, CD40L, and PKCβ associate following activation of CD40L reverse signaling in hippocampal pyramidal neurons. PKCγ, the PKC isoform required for the effects of CD40L reverse signaling on dendrite growth from medial spiny neurons [18], does not appear to be a component of the CD40L receptor complex in hippocampal pyramidal neurons. These data are consistent with extensive evidence that Syk and PKC are recruited to the membrane following activation of relevant receptors [33, 35]. Interestingly, T-lymphocyte activation via CD40L involves recruitment of p56lck and PKC to the membrane and is associated with strong activation of JNK [37, 38]. p56lck is a non-receptor tyrosine kinase that is structurally and functionally related to Syk [39].

In addition to Syk, in silico analysis suggests that components of JNK, ERK, and ERK-activating enzymes associate with CD40L and PKCβ. Thus, it is possible that activation of CD40L reverse signaling initiates the formation of a receptor complex that includes all of intracellular signaling elements we have studied. The physical interaction of these components may explain the functional interactions between PKC, ERK, and JNK that mediate and determine the consequences of activation of CD40L reverse signaling in hippocampal pyramidal neurons. Future work may reveal how such potential physical interactions might generate the complex interdependencies between PKC, ERK, and JNK that our work has revealed.

## Electronic supplementary material

Below is the link to the electronic supplementary material.

**Expression of Syk, PKCβ, PKCγ and CD40L. a** Representative Western blot of the expression of Syk, PKCβ, PKCγ and CD40L in hippocampal tissue from* Cd40*^+/+^ and* Cd40*^-/-^ at the indicated ages.** b** Representative Western blot of the expression of CD40L and CD40 in hippocampal neurons from* Cd40*^+/+^ and* Cd40*^-/-^ cultured for the days indicated.** c** Representative Western blot of the expression of Syk, PKCβ, PKCγ and CD40L from* Cd40*^-/-^ hippocampal neurons cultured in vitro the days indicated. Anti-βIII tubulin was used as a loading control. * = non specific band. (PDF 3202 kb)

**Total neuronal length (axon + dendrites).** Quantification of total axon (grey) and dendrite (white) lengths of neurons cultured for 9 days in vitro, and treated 24 h after plating with the indicating combination of reagents in presence of 1 μg/ml CD40-Fc. Control Fc at 1μg/ml. The graph shows the mean ± s.e.m of at least three independent experiments. T-test comparisons versus neurons treated with Fc, *** p < 0.001, ** p < 0.01 and * p < 0.05. (PDF 2731 kb)

Protein-protein interactions (PPI) for mouse CD40L and PKCβ and in common between CD40L and PKCβ. (PDF 204 kb)

Protein-protein interactions (PPI) shared between CD40L and PKCβ relevant for CD40-activated CD40L reverse signaling. (PDF 459 kb)
