# Supplementary figures and images for: How CD40L reverse signaling regulates axon and dendrite growth
Source: Cell Mol Life Sci. 2020 Jun 6;78(3):1065–83. doi: 10.1007/s00018-020-03563-2 (PMC7897621; doi:10.1007/s00018-020-03563-2)

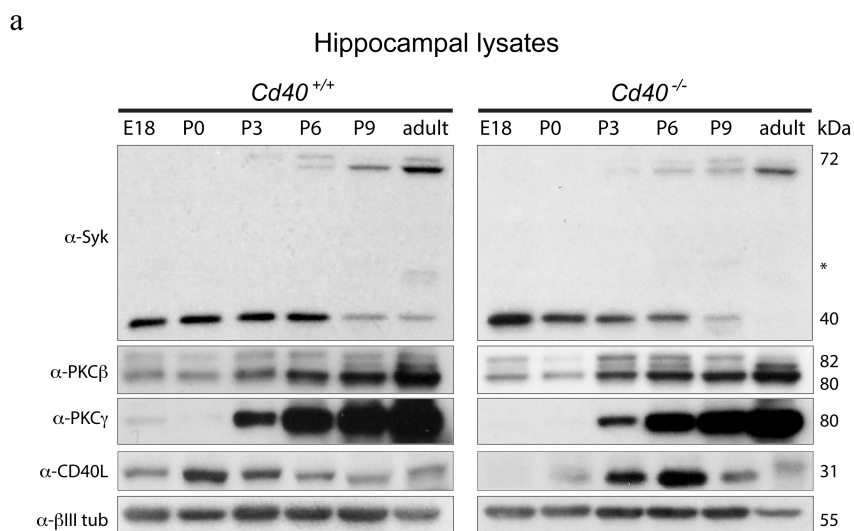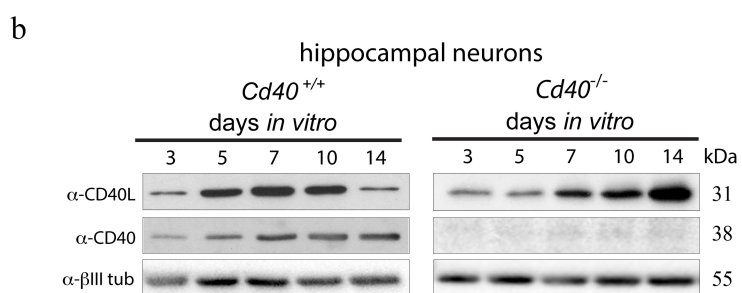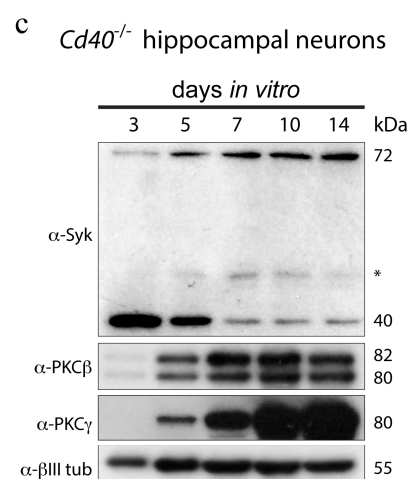

Supplement: Supplementary file 1 — Expression of Syk, PKCβ, PKCγ and CD40L. a Representative Western blot of the expression of Syk, PKCβ, PKCγ and CD40L in hippocampal tissue from Cd40+/+ and Cd40-/- at the indicated ages. b Representative Western blot of the expression of CD40L and CD40 in hippocampal neurons from Cd40+/+ and Cd40-/- cultured for the days indicated. c Representative Western blot of the expression of Syk, PKCβ, PKCγ and CD40L from Cd40-/- hippocampal neurons cultured in vitro the days indicated. Anti-βIII tubulin was used as a loading control. * = non specific band. (PDF 3202 kb) [file 18_2020_3563_MOESM1_ESM.pdf]

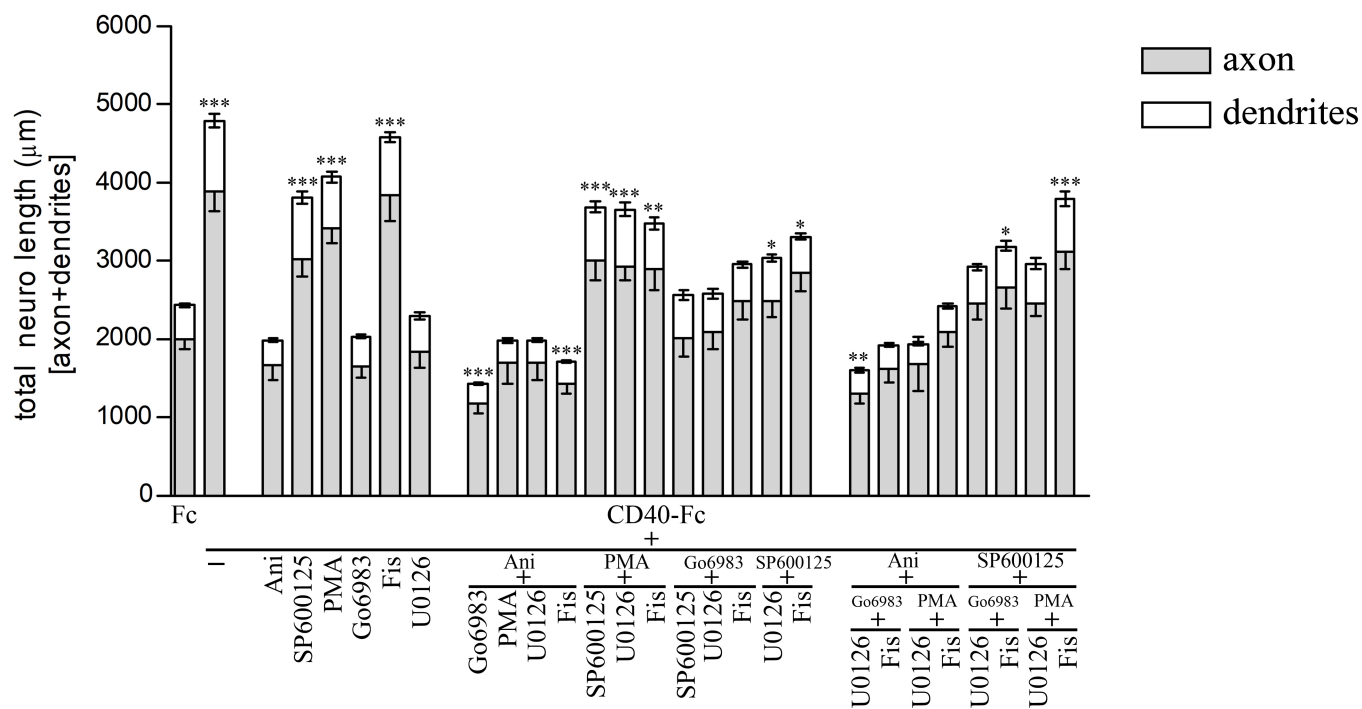

Supplement: Supplementary file 2 — Total neuronal length (axon + dendrites). Quantification of total axon (grey) and dendrite (white) lengths of neurons cultured for 9 days in vitro, and treated 24 h after plating with the indicating combination of reagents in presence of 1 μg/ml CD40-Fc. Control Fc at 1μg/ml. The graph shows the mean ± s.e.m of at least three independent experiments. T-test comparisons versus neurons treated with Fc, *** p < 0.001, ** p < 0.01 and * p < 0.05. (PDF 2731 kb) [file 18_2020_3563_MOESM2_ESM.pdf]
