# Supplementary material for: How CD40L reverse signaling regulates axon and dendrite growth
Source: Cell Mol Life Sci. 2020 Jun 6;78(3):1065–83. doi: 10.1007/s00018-020-03563-2 (PMC7897621; doi:10.1007/s00018-020-03563-2)
Supplement: Supplementary file 3 — Protein-protein interactions (PPI) for mouse CD40L and PKCβ and in common between CD40L and PKCβ. (PDF 204 kb) [file 18_2020_3563_MOESM3_ESM.pdf]

Predicted Functional Partners

CD40L		PKCb		in common	CD40L	PKCb
Cd40	0,999	Pdpk1	0,977	Bcl2	0,609	0,41
Tnf	0,977	Hras1	0,966	Casp3	0,43	0,521
Traf3	0,975	Kras	0,965	Cd38	0,635	0,824
Traf2	0,97	Sphk1	0,951	Fos	0,65	0,88
Birc2	0,969	Prkcz	0,95	Fyn	0,47	0,425
Tnfsf13b	0,964	Mtor	0,95	Icam1	0,856	0,477
Lta	0,959	Prkcd	0,942	Icam5	0,511	0,407
Tnfrsf13c	0,944	Gnb1	0,929	Il2	0,901	0,755
Map3k14	0,94	Card11	0,928	Itgb1	0,543	0,859
Tnfrsf1b	0,938	Mapk1	0,927	Itgb3	0,537	0,922
Ltbr	0,931	Plcg2	0,927	Itgb7	0,466	0,848
Ltb	0,929	Mapk3	0,926	Jak1	0,566	0,597
Tnfsf12	0,928	Raf1	0,926	Jak2	0,596	0,527
Tnfrsf11a	0,925	Ap2a1	0,926	Jak3	0,704	0,593
Tnfsf11	0,918	Braf	0,926	Lck	0,59	0,621
Tnfsf14	0,916	Ap2a2	0,925	Lepr	0,623	0,429
Psmd10	0,915	Itgb3	0,922	Lyn	0,547	0,885
Psma1	0,913	Plcg1	0,921	Map2k4	0,573	0,401
Cd86	0,914	Dvl2	0,921	Map3k1	0,581	0,528
Psmb8	0,913	Malt1	0,917	Map3k14	0,94	0,428
Tnfrsf12a	0,913	Stxbp3a	0,917	Map3k5	0,478	0,532
Il4	0,913	Gria1	0,916	Mapk1 (ERK2)	0,529	0,927
Psmb10	0,908	Bcl10	0,916	Mapk10 (JNK3)	0,561	0,555
Il2	0,901	Gria4	0,915	Mapk11	0,45	0,551
Psma6	0,901	Gria3	0,915	Mapk14	0,674	0,561
Psmd5	0,9	Ap2m1	0,911	Mapk3 (ERK1)	0,592	0,926
Psmc6	0,9	Nras	0,911	Mapk8 (JNK1)	0,678	0,558
Psmb6	0,9	Lmna	0,909	Mapk9 (JNK2)	0,593	0,553
Gm21972	0,9	Fzd4	0,908	Nfkb1	0,778	0,593
Gm5422	0,9	Stx4a	0,907	Nfkb2	0,595	0,458
Psme1	0,9	Grip1	0,906	Nfkbia	0,679	0,658
Psmb11	0,9	Rhoa	0,905	Nos1	0,535	0,687
Psma2	0,9	Prkcg	0,904	Rel	0,734	0,436
Psma3	0,9	Rasa1	0,904	Rela	0,74	0,595
Psmd4	0,9	Wnt5a	0,904	Sele	0,702	0,477
Psmb3	0,9	Ap2b1	0,901	Spna2	0,54	0,448
Psmd9	0,9	Gngt1	0,901	Src	0,536	0,477
Gm4950	0,9	Ap2s1	0,901	Stat5a	0,66	0,522
Psma5	0,9	Emd	0,9	Stat5b	0,504	0,507
Gm8394	0,9	Itgb4	0,899	Syk	0,528	0,439
Psmc3	0,9	Rras	0,898	Tnfsf11	0,918	0,452
Psmd8	0,9	Adcy9	0,898	Trp53	0,495	0,529
Psme4	0,9	Rras2	0,894	Tyk2	0,497	0,446

Psma8	0,9	Gnai1	0,892	Vcam1	0,62	0,464
Psmd7	0,9	Gnao1	0,892	Xiap	0,605	0,433
Psmf1	0,9	Atf2	0,891			
Psma4	0,9	Rhob	0,89			
Psmc4	0,9	Rhoc	0,889			
Psmb2	0,9	Gnb4	0,887			
Psma7	0,9	Irs1	0,887			
Psmd14	0,9	Adcy5	0,886			
Psmb7	0,9	Adcy2	0,886			
Psmd1	0,9	Lyn	0,885			
Psmd13	0,9	Gnai2	0,882			
Psmb5	0,9	Gnai3	0,882			
Psmd6	0,9	Prkcq	0,882			
Psmc1	0,9	Gnb2	0,881			
Psmd12	0,9	Gnb3	0,881			
Psmc5	0,9	Adcy1	0,881			
Psme3	0,9	Fos	0,88			
Psmd11	0,9	Adcy3	0,88			
Psmd3	0,9	Adcy7	0,878			
Psmb1	0,9	Mras	0,878			
Psmd2	0,9	Ptk2	0,877			
Psmb4	0,9	Adcy8	0,877			
Icos	0,882	Plcb1	0,874			
Cd83	0,875	Adcy6	0,873			
Cd80	0,874	Adcy4	0,873			
Il6	0,874	mCG_125315	0,873			
Cd79a	0,867	Insr	0,873			
Itgax	0,863	Ppp2ca	0,87			
Icam1	0,856	Creb1	0,87			
Traf1	0,844	Sphk2	0,868			
Akt1	0,842	Gng11	0,866			
Itgam	0,837	Itgb8	0,866			
Il15	0,832	Ncf1	0,864			
Cd69	0,821	Itgb1	0,859			
Il2ra	0,821	Plcb3	0,858			
Il12b	0,817	Ppp2cb	0,853			
Ctla4	0,816	Plcd1	0,853			
Foxp3	0,807	Creb5	0,853			
Fasl	0,805	Plcb4	0,85			
Fas	0,805	Itgb7	0,848			
Ddx41	0,802	Cacna1a	0,848			
Il10	0,797	Pla2g4a	0,847			
Csf2	0,797	Itgb5	0,847			
Cd19	0,797	Plcb2	0,847			
Tnfsf4	0,796	Gng7	0,843			
Cd276	0,782	Plcd3	0,842			

Ifng	0,78	Plce1	0,842
Traf6	0,779	Gng3	0,841
Nfkb1	0,778	Eef2k	0,84
Ccl5	0,773	Plcd4	0,84
Nfatc3	0,772	Ncf2	0,84
Icosl	0,764	Itpr1	0,839
Cd1d1	0,763	Itgb6	0,838
Jun	0,759	Ppp2r1a	0,835
Rela	0,74	Ezr	0,834
Rel	0,734	Plcz1	0,833
F3	0,735	Prkd1	0,832
Cd27	0,733	Gng2	0,831
Bcl2l1	0,72	Gng4	0,831
Bcl6	0,712	Gng13	0,83
Cd28	0,709	Gngt2	0,83
Il5	0,705	Gm3150	0,829
Jak3	0,704	Gm5741	0,829
Cd209a	0,703	Gng8	0,829
Sele	0,702	Gng12	0,829
Ccr7	0,702	Gng10	0,829
Irf4	0,7	Pla2g6	0,829
Tnfrsf4	0,7	Ppp2r1b	0,826
Mboat4	0,699	Ttf2	0,825
Birc3	0,698	Cd38	0,824
Nfatc2	0,698	Pla2g4e	0,823
Ido1	0,697	Pla2g4f	0,823
Cxcl12	0,695	Slc9a1	0,82
Nfatc1	0,683	Pla2g4c	0,82
Nfkbia	0,679	Pla2g4d	0,818
Ptprc	0,678	Pla2g4b	0,818
Mapk8	0,678	Prkd2	0,815
Stat6	0,675	Prkd3	0,814
Mapk14	0,674	Gnb5	0,813
Itgb2	0,671	F11r	0,813
Sell	0,668	Itpr2	0,813
Cd5	0,665	Itpr3	0,813
Il13	0,665	Ncf4	0,813
Cd70	0,665	Tjp1	0,81
Tlr4	0,664	Prkca	0,81
Prf1	0,663	Tyr	0,81
Tlr9	0,663	Trpv4	0,807
Tnfrsf1a	0,662	Rac2	0,806
H2-K1	0,662	Slc6a3	0,805
Il7	0,661	Ttf1	0,804
Stat5a	0,66	Atf6b	0,803
Alb	0,658	Creb3l2	0,803

Fos	0,65	Creb3l3	0,802
Crp	0,643	Creb3l4	0,802
Ccl2	0,643	Egln2	0,802
Bcr	0,642	Egln1	0,802
Tgfb1	0,642	Atf4	0,802
Cd38	0,635	Pax8	0,802
C3	0,633	Creb3	0,801
Il18	0,627	Creb3l1	0,801
Cd44	0,626	Egln3	0,8
Ccr5	0,624	Jam2	0,8
H2-D1	0,623	Igsf5	0,8
Lepr	0,623	Jam3	0,8
Vcam1	0,62	Atp6ap1	0,8
Tnfrsf10b	0,62	Cdc42	0,796
Cxcl15	0,619	Chuk	0,792
Ncam1	0,617	Rac1	0,784
Stat1	0,614	Rac3	0,773
Selp	0,613	Il2	0,755
Fcgr2b	0,611	Gnb2l1	0,719
Cd34	0,611	Nos1	0,687
Tnfrsf9	0,61	Rictor	0,677
Klrb1c	0,609	Marcks	0,676
Bcl2	0,609	Pld2	0,67
Tnfsf9	0,608	Shc1	0,67
Il17a	0,607	Nfkbia	0,658
Tlr7	0,607	Pld1	0,647
Cxcr5	0,607	Ikbkb	0,645
Anxa5	0,606	Rb1	0,644
Thy1	0,606	Phlpp1	0,639
H2-Ab1	0,606	Calm3	0,634
Ly75	0,606	Decr1	0,632
Tnfsf8	0,605	Rhobtb2	0,631
Xiap	0,605	Pi4ka	0,629
Hand2	0,604	Rhoh	0,627
Tlr2	0,603	Hsp90ab1	0,624
Cd14	0,603	Smg1	0,623
Il23a	0,603	Lck	0,621
Tnfrsf8	0,602	Egf	0,621
Stat3	0,601	Pdk2	0,617
Relb	0,601	Ppp1r14a	0,617
Il3ra	0,601	Phlpp2	0,617
Serpinb1a	0,601	Hsp90aa1	0,616
Flt3	0,598	Prkdc	0,613
Jak2	0,596	Ptk2b	0,612
Nfkb2	0,595	Calm1	0,612
Mapk9	0,593	Rhoj	0,606

Mapk3	0,592	Lrrk2	0,606
Lck	0,59	Egfr	0,602
Map3k1	0,581	Rhog	0,599
Map2k4	0,573	Rhov	0,599
Cblb	0,571	Rhou	0,599
ENSMUSG00000095585	0,57	Rhod	0,599
Jak1	0,566	Rhof	0,599
Mapk10	0,561	Rhoq	0,599
Prdm10	0,558	Rhobtb3	0,599
Fcgr3	0,554	Rhobtb1	0,599
Serpinb6c	0,55	Rnd3	0,599
Serpinb6b	0,55	Rnd1	0,599
Serpinb6e	0,55	Rnd2	0,599
Serpinb7	0,55	Ripk4	0,598
Serpinb9g	0,55	Jak1	0,597
Serpinb9f	0,55	Rela	0,595
Serpinb9e	0,55	Actb	0,595
Cxcl10	0,55	Jak3	0,593
Serpinb3c	0,55	Nfkb1	0,593
Serpinb9c	0,55	Mapkap1	0,59
Serpinb1c	0,55	Lrrk1	0,59
Cd274	0,55	Actg1	0,588
Serpinb9b	0,55	Fras1	0,583
Serpinb9	0,55	Ppp3ca	0,583
Serpinb8	0,55	Map2k1	0,579
Btk	0,549	Pik3c3	0,579
Itga2b	0,549	Araf	0,578
Dct	0,549	Rps6kb1	0,577
Serpinb6a	0,549	Actc1	0,574
Serpinb1b	0,549	Actg2	0,574
Serpinb2	0,549	Actbl2	0,574
Gapdh	0,548	Acta2	0,574
Serpinb9d	0,548	Acta1	0,574
Lyn	0,547	Erbb4	0,573
Alpi	0,547	Inpp5d	0,571
Myd88	0,546	Pik3cg	0,57
Zap70	0,546	Ldb3	0,568
Aicda	0,545	Ppp3cb	0,567
Serpinb5	0,545	Shank3	0,567
Cxcr3	0,543	Mapk14	0,561
Map4k4	0,543	Elk1	0,56
Itgb1	0,543	Mapk8	0,558
Gm2023	0,541	Nrg3	0,557
Spna2	0,54	Mapk10	0,555
Il12a	0,54	Rptor	0,554
H2-Q2	0,54	Mapk9	0,553

Itgb3	0,537	Rps6	0,552
Tdp2	0,536	Mapk11	0,551
Src	0,536	Map2k2	0,55
Ccl19	0,536	Keap1	0,548
Tlr3	0,536	Adam12	0,546
Nos1	0,535	Pik3ca	0,546
Cxcr4	0,531	Rem1	0,545
Nfatc4	0,529	Adam17	0,545
Mapk1	0,529	Gnas	0,544
Syk	0,528	Wdr31	0,544
Cbl	0,527	Cabin1	0,542
Ccl11	0,525	Gnaq	0,54
Mmp11	0,524	Mapk12	0,538
Itga4	0,516	Mapk13	0,538
Rag1	0,515	Mapk4	0,538
Ms4a1	0,512	Pik3cb	0,537
Cr2	0,512	Mapk7	0,535
Icam5	0,511	Pik3cd	0,534
Ccl3	0,51	Gm20489	0,532
Birc5	0,508	Il2rg	0,532
F2	0,508	Map3k5	0,532
Stat5b	0,504	Trp53	0,529
Nr3c1	0,505	Map3k1	0,528
Tnfrsf13b	0,504	Map3k6	0,527
Cdkn1a	0,504	Jak2	0,527
Ccr2	0,502	Tfeb	0,527
Il7r	0,501	Ppp3cc	0,526
Cd22	0,499	Map3k15	0,525
Tyk2	0,497	Tep1	0,524
Rag2	0,496	Stat5a	0,522
Sdc1	0,496	Casp3	0,521
Trp53	0,495	Prkacb	0,52
Tnfrsf18	0,489	Akap5	0,515
Ighg2c	0,488	Cabp1	0,515
Emr1	0,485	Hck	0,513
Myh1	0,484	Prkci	0,513
Tbx21	0,483	Caln1	0,511
Map3k5	0,478	Fbxw7	0,509
Spn	0,476	Stat5b	0,507
Gzmb	0,475	Ocln	0,508
Csf3	0,473	Ywhab	0,506
Fyn	0,47	Mapk6	0,502
Il1b	0,47	Mapk15	0,5
Nfkbib	0,469	Sdc4	0,497
Il4ra	0,468	Dlg4	0,497
Cd68	0,468	Vegfa	0,494

Cd4	0,467
Itgb7	0,466
Cxcl9	0,465
Fcgr4	0,462
Flt3l	0,461
Pdcd1lg2	0,454
Cast	0,453
Map2k6	0,453
Cyth1	0,452
Mapk11	0,45
Apoe	0,45
Prdm1	0,448
Atp7a	0,448
Cxcl13	0,447
Il9	0,447
Cd207	0,445
Map2k3	0,443
Itga2	0,443
Ccl4	0,443
Wdfy2	0,442
mt-Nd1	0,442
Il21	0,442
Csf1	0,442
Dnase1	0,442
Npr2	0,44
Faim3	0,44
Vwf	0,438
Slc11a1	0,438
Cd48	0,437
Irak1	0,436
Itih4	0,436
Pmel	0,434
Bcl3	0,433
Lamp3	0,432
Stat2	0,432
Tlr1	0,432
Rassf5	0,432
Casp3	0,43
Ptgs2	0,43
Tmprss11d	0,43
Traf5	0,429
Kit	0,429
Mmp9	0,429
Casp8	0,428
Apoh	0,427
Ccr1	0,425

Mlst8	0,492
Prkaca	0,491
Calm2	0,491
Rps6kb2	0,49
Arhgef3	0,489
Gnal	0,487
Map2k5	0,482
Ywhaz	0,481
Ywhae	0,479
Ywhah	0,479
Prkx	0,477
Igf1	0,477
Sele	0,477
Icam1	0,477
Src	0,477
Calb1	0,477
Net1	0,474
Cish	0,473
Gm16505	0,472
ENSMUSG00000055657	0,472
Pvalb	0,472
Zfand4	0,471
Srebf1	0,47
Tnnc2	0,469
Tnnc1	0,468
Cabp2	0,467
Gm11639	0,466
Efcab6	0,466
Efcab7	0,466
Calml3	0,466
Efcab9	0,466
Spata21	0,466
Efcab11	0,466
Calm4	0,466
Calml4	0,466
Ocm	0,466
Efcab2	0,466
Cabp4	0,466
Scgn	0,466
ENSMUSG00000020940	0,466
Efcab3	0,466
Cabp7	0,466
Cabp5	0,466
Calb2	0,466
Ywhag	0,466
Ret	0,465

Il2rb	0,424
Mme	0,424
Eif6	0,422
Ccr3	0,422
Pdcd1	0,422
Itgal	0,42
Slamf1	0,42
Itgae	0,42
Stat4	0,42
C4b	0,409
Kitl	0,409
Cntnap1	0,408
Tlr8	0,407
Akt3	0,407
Mbp	0,407
Selplg	0,405
Il10ra	0,404
Cd36	0,404
Vamp8	0,404
Tslp	0,404
Akt2	0,403
Cd8a	0,402
Il21r	0,402
Fcgr1	0,401
Gata3	0,4

Vcam1	0,464
Edn1	0,464
Cdh5	0,464
Actr1b	0,462
Grin2b	0,463
Pfkfb3	0,461
Pfkfb2	0,461
Pik3c2a	0,46
Pik3c2g	0,46
Trrap	0,46
Atr	0,46
ENSMUSG00000096062	0,46
Ywhaq	0,46
Sfn	0,46
Tbxa2r	0,459
Gfpt1	0,459
Nfkb2	0,458
Actr1a	0,456
Nos2	0,455
Ehmt1	0,454
Ehmt2	0,454
Nos3	0,453
Tnfsf11	0,452
Grin1	0,452
Spna2	0,448
Tert	0,448
Tyk2	0,446
Gna11	0,445
Diras2	0,444
Actl11	0,442
Atf7	0,441
Rhbdl3	0,441
Mef2d	0,441
Rasl10b	0,441
Grb2	0,441
Snca	0,441
Actl9	0,44
Gm498	0,44
Actl10	0,44
Actl7b	0,44
Actl7a	0,44
Actrt1	0,44
Actrt2	0,44
Arpm1	0,44
Actr10	0,44
Mef2a	0,44

Pdk1	0,439
Prkg2	0,439
Rasd2	0,439
Syk	0,439
Pfkfb1	0,437
Pfkfb4	0,437
Mylpf	0,437
Gnat2	0,437
Gnat3	0,437
Gnat1	0,437
Yes1	0,437
Gfpt2	0,436
Rel	0,436
Prkg1	0,435
Cd8b1	0,435
Ppp2r5c	0,435
Cnot6l	0,434
Cnot6	0,434
Xiap	0,433
Cdk17	0,432
Pik3c2b	0,43
Inppl1	0,43
Gna13	0,43
Gna12	0,43
Cdk2	0,429
Lepr	0,429
Map3k14	0,428
Prkar2b	0,428
Bad	0,427
Rap1b	0,426
Cdk1	0,425
Gna14	0,425
Fyn	0,425
Crk	0,424
Ysk4	0,423
Map3k2	0,423
Map3k3	0,423
Rasgrp1	0,421
Diras1	0,421
Gnaz	0,419
Mef2c	0,419
Itsn1	0,419
Itsn2	0,419
Rap2a	0,418
Vav1	0,419
Lrdd	0,418

Adam33	0,416
Adam19	0,416
Sod2	0,414
Cacna1e	0,415
Gna15	0,413
Myc	0,413
Ppp2r5d	0,413
Ppp2r5b	0,413
Pdk3	0,412
Pdk4	0,412
Arf3	0,412
Bcl2	0,41
Pik3r1	0,41
Pak1	0,411
Prkar1b	0,409
Mef2b	0,408
ENSMUSG00000002345	0,408
Irs2	0,408
Blk	0,408
Cdk16	0,408
Icam5	0,407
Nwd1	0,407
Bcar1	0,406
Nedd9	0,406
Sos1	0,405
Eif4ebp1	0,405
Arhgef10l	0,403
Umps	0,403
Cdk5	0,402
Stt3a	0,402
Stt3b	0,402
Pip5k1c	0,402
Map2k4	0,401
Gdnf	0,401
Tsc2	0,4
Ppp2r5e	0,4
