# Supplementary material for: How CD40L reverse signaling regulates axon and dendrite growth
Source: Cell Mol Life Sci. 2020 Jun 6;78(3):1065–83. doi: 10.1007/s00018-020-03563-2 (PMC7897621; doi:10.1007/s00018-020-03563-2)
Supplement: Supplementary file 4 — Protein-protein interactions (PPI) shared between CD40L and PKCβ relevant for CD40-activated CD40L reverse signaling. (PDF 459 kb) [file 18_2020_3563_MOESM4_ESM.pdf]

	Score Predicted PPI	Score Predicted PPI
	**CD40L**	**PKCβ**
**Mapk8 (Jnk1)**	**0.678**	**0.558**
**Mapk9 (Jnk2)**	**0.593**	**0.553**
**Mapk3 (ERK 1)**	**0.592**	**0.926**
**Map3k1**	**0.581**	**0.528**
**Map2k4**	**0.573**	**0.401**
**Mapk10 (Jnk3)**	**0.561**	**0.555**
**Mapk1 (ERK 2)**	**0.529**	**0.927**
**Syk**	**0.528**	**0.439**
**Map3k5**	**0.478**	**0.532**

Map3k1: Activates ERK and JNK kinase pathways
Map2k4: Essential component of the MAPK and JNK signal transduction pathways
Syk: Spleen tyrosine kinase. Mediates signal transduction downstream of a variety of transmembrane receptors
Map3k5: Activates JNK, but it does not activate MAPK/ERK
